# TRAF7 is an essential regulator of blood vessel integrity during mouse embryonic and neonatal development

**DOI:** 10.1016/j.isci.2023.107474

**Published:** 2023-07-25

**Authors:** Erdyni N. Tsitsikov, Khanh P. Phan, Yufeng Liu, Alla V. Tsytsykova, Mike Kinter, Lauren Selland, Lori Garman, Courtney Griffin, Ian F. Dunn

**Affiliations:** 1 Department of Neurosurgery, University of Oklahoma Health Sciences Center, Oklahoma City, OK 73104, USA; 2 Aging & Metabolism Research Program, Oklahoma Medical Research Foundation, Oklahoma City, OK 73104, USA; 3 Histology, Immunohistochemistry, Microscopy Core-COBRE Stephenson Cancer Center, University of Oklahoma Health Sciences Center, Oklahoma City, OK 73104, USA; 4 Department of Microbiology and Immunology, University of Oklahoma Health Sciences Center, Oklahoma City, OK 73104, USA; 5 Cardiovascular Biology Research Program, Oklahoma Medical Research Foundation, Oklahoma City, OK 73104, USA

**Keywords:** Molecular biology, Developmental biology, Omics, Transcriptomics

## Abstract

Targeted deletion of TRAF7 revealed that it is a crucial part of shear stress-responsive MEKK3-MEK5-ERK5 signaling pathway induced in endothelial cells by blood flow. Similar to *Mekk3-*, *Mek5-* or *Erk5*-deficient mice, *Traf7*-deficient embryos died *in utero* around midgestation due to impaired endothelium integrity. They displayed significantly lower expression of transcription factor *Klf2*, an essential regulator of vascular hemodynamic forces downstream of the MEKK3-MEK-ERK5 signaling pathway. In addition, deletion of *Traf7* in endothelial cells of postnatal mice was associated with severe cerebral hemorrhage. Here, we show that besides MEKK3 and MEK5, TRAF7 associates with a planar cell polarity protein SCRIB. SCRIB binds with an N-terminal region of TRAF7, while MEKK3 associates with the C-terminal WD40 domain. Downregulation of TRAF7 as well as SCRIB inhibited fluid shear stress-induced phosphorylation of ERK5 in cultured endothelial cells. These findings suggest that TRAF7 and SCRIB may comprise an upstream part of the MEKK3-MEK5-ERK5 signaling pathway.

## Highlights

• *Traf7* deletion results in embryonic lethality
• TRAF7 is important for KLF2 expression
• In addition to MEKK3 and MEK5, TRAF7 binds SCRIB
• TRAF7 and SCRIB participate in ERK5 phosphorylation in endothelial cells

## Graphical abstract

**Figure:**
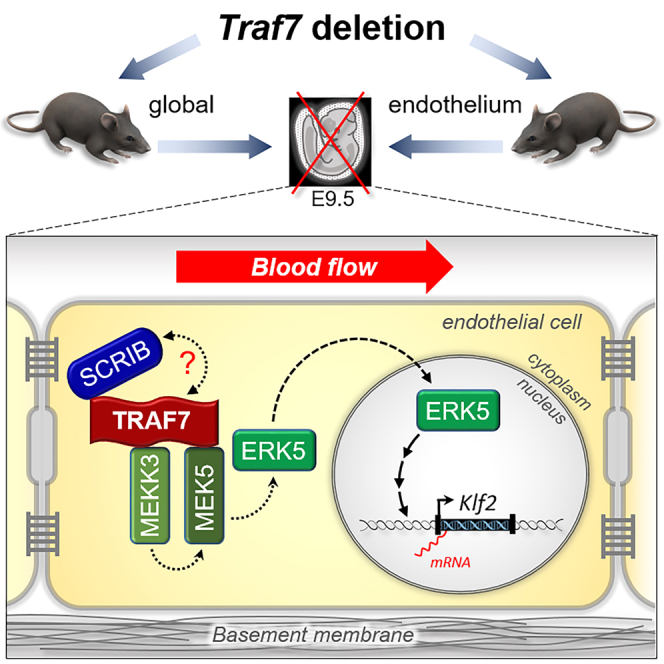

## Introduction

The TRAF (TNF receptor-associated factor) family consists of seven adaptor proteins with diverse roles in intracellular signaling.^1^ While TRAF1 to TRAF6 are well-characterized molecules involved in immunity and inflammation, TRAF7 function remains unclear. TRAF7 was shown to be mutated in 30% of meningiomas, the most common primary tumor of the central nervous system (CNS).^2^^,^^3^^,^^4^ In addition, *de novo* mutations in *Traf7* gene were described in patients with developmental delay, congenital heart defects, limb and digital anomalies, and dysmorphic features.^5^^,^^6^ All reported somatic as well as germline *Traf7* mutations are hemizygous missense and fall into different positions within the C-terminal part of the protein, suggesting gain-of-function and/or dominant-negative effects of the resulting mutant protein, rather than loss of function resulting in haploinsufficiency. Although almost all identified mutations are recurrent (the same in different tumors or patients), somatic and germline mutations do not overlap. For example, while a germline R655Q mutation appears in almost 30% of patients with TRAF7 syndrome,^5^ no published meningiomas have a mutated R655, implying that signaling programs initiated by somatic versus germline TRAF7 variants may be different and may direct significantly divergent courses of action.

All mammalian TRAFs, with the exception of TRAF1, contain a characteristic N-terminal RING (really interesting new gene) finger domain, several adjacent zinc fingers (ZFs), and a coiled coil (CC) motif, which fosters formation of TRAF trimers.^1^ At the C terminus, six TRAFs (1 through 6) carry a conserved TRAF domain responsible for binding to other proteins, including cell surface receptors and intracellular adapters.^7^ Unlike the rest of the family members, TRAF7 lacks the TRAF domain and instead contains a tryptophan-aspartic acid (W-D) dipeptide (WD40) domain consisting of seven WD40 repeats. For that reason, TRAF7 is often placed into a RING finger and WD domain (RFWD) family of proteins and is called RFWD1. This group also includes RFWD2, an E3 ubiquitin ligase constitutive photomorphogenic 1 (COP1),^8^^,^^9^ and RFWD3.^10^ Importantly, the structure of COP1 and RFWD3 RING fingers is very different from that of the canonical TRAF-family RING domain of TRAF7. Thus, it is not surprising that a recent phylogenetic analysis assigned TRAF7 into a separate group that developed independently from other TRAFs throughout vertebrate evolution.^11^ In fact, unlike other TRAFs, TRAF7 remained highly conserved during evolution; humans and lampreys (*Petromyzon marinus*) share more than 80% of the whole protein and 93% of the WD40 domain amino acid (aa) sequences. Thus, TRAF7 is a unique protein without close paralogs in vertebrates.

Over the years, TRAF7 has been shown to participate in different signaling pathways, ranging from activation of nuclear factor kappa-light-chain-enhancer of activated B cells (NF-κB) to Activator protein 1 (AP-1),^12^ and to suppress endothelial cell hyperpermeability in inflammation.^13^ Two decades ago TRAF7 was discovered as a part of a mitogen-activated protein kinase kinase kinase 3 (MAP3K3, also known as MEKK3)-associated protein complex using tandem affinity purification.^12^ This complex also included dual specificity mitogen-activated protein kinase kinase 5 (MAP2K5, also known as MEK5), serine/threonine-protein kinase MARK2 (also known as EMK1), prefoldin 2 (PFDN2), heat shock protein 70 (HSP70), and p53 and DNA damage regulated 1 (PDRG1, also known as C20orf126).^12^ In the same year, Xu et al. independently confirmed the association of TRAF7 with MEKK3^14^ and suggested that TRAF7 is important for MEKK3-mediated activation of AP-1 and DNA damage-inducible transcript 3 protein (DDIT3, also known as CHOP). TRAF7 was also shown to associate with a number of other molecules, including cylindromatosis 1 (CYLD1),^15^ transcriptional activator c-MYB,^16^ myoblast determination protein 1 (MYOD1),^17^ NF-kappa-B essential modulator (NEMO),^18^ and roundabout homolog 4 (ROBO4).^13^ A recent high-throughput affinity-purification mass spectrometry interactome study demonstrated that TRAF7 interacts with MEK5 and indirectly with MEKK3.^19^ In addition, TRAF7, together with cerebral cavernous malformations 1 (CCM1, also known as KRIT1) and CCM2, precipitated with MEKK3, which was necessary and sufficient for CCM-dependent expression of the shear stress-responsive transcription factors Krüppel-like factor 2 (KLF2) and its close paralog KLF4.^20^

MAPK signaling pathways are activated by a wide variety of stimuli such as mitogens, osmotic stress, and pro-inflammatory cytokines. Each MAPK cascade consists of at least three enzymes that are activated in chains following phosphorylation: a MAPK kinase kinase (MAP3K), a MAPK kinase (MAP2K), and a MAP kinase (MAPK). MEKK2 and MEKK3 are unique among MAP3K kinases because each of them possesses an N-terminal PB1 (Phox and Bem1) domain, which is used to bind to PB1 domain of MEK5.^21^ Following hetero-dimerization with MEK5, MEKK2 or MEKK3 phosphorylates and activates MEK5 in response to different stimuli, including blood flow shear stress.^21^ In turn, activated MEK5 phosphorylates and activates extracellular signal-regulated kinases 5 (ERK5, also known as MAPK7 and Big MAPK Kinase 1 [BMK1]), which was originally identified as a MAPK activated by both osmotic and oxidative stress.^22^ Interestingly, ERK5 is also unique among MAPKs due to the presence of a C-terminal extension, which gives the protein approximately twice the molecular weight of other MAPKs. The MEK5/ERK5 pathway is known to be activated by shear stress and to sustain resistance of endothelial cells to apoptosis.^23^^,^^24^ This pathway is also important for shear stress-dependent induction of KLF2.^25^^,^^26^^,^^27^^,^^28^

Despite significant progress in understanding of the role of MEKK3-MEK5-ERK5-KLF2 pathway in endothelial cells, the upstream mechanism of shear-stress signal transduction is still unclear. Here, we generated *Traf7*-deficient mice to elucidate the physiological role of TRAF7. Global *Traf7* deletion resulted in embryonic lethality. The *Traf7*^*−/−*^ embryos failed to develop beyond E10 due to impaired blood vessel integrity. A detailed pathology analysis revealed evidence of intraembryonic hemorrhage and hypoxia. Transcriptome analysis of failing embryos demonstrated significantly low expression of transcription factor KLF2 among other genes. We also show that besides MEKK3 and MEK5 TRAF7 associates with a planar cell polarity protein scribble homolog (SCRIB) and that downregulation of TRAF7 as well as SCRIB inhibited fluid shear stress-induced phosphorylation of ERK5. Thus, TRAF7 is a part of MEKK3-MEKK5-ERK5 signaling pathway.

## Results

### Targeted deletion of *Traf7* gene causes embryonic lethality

**Figure 1:**
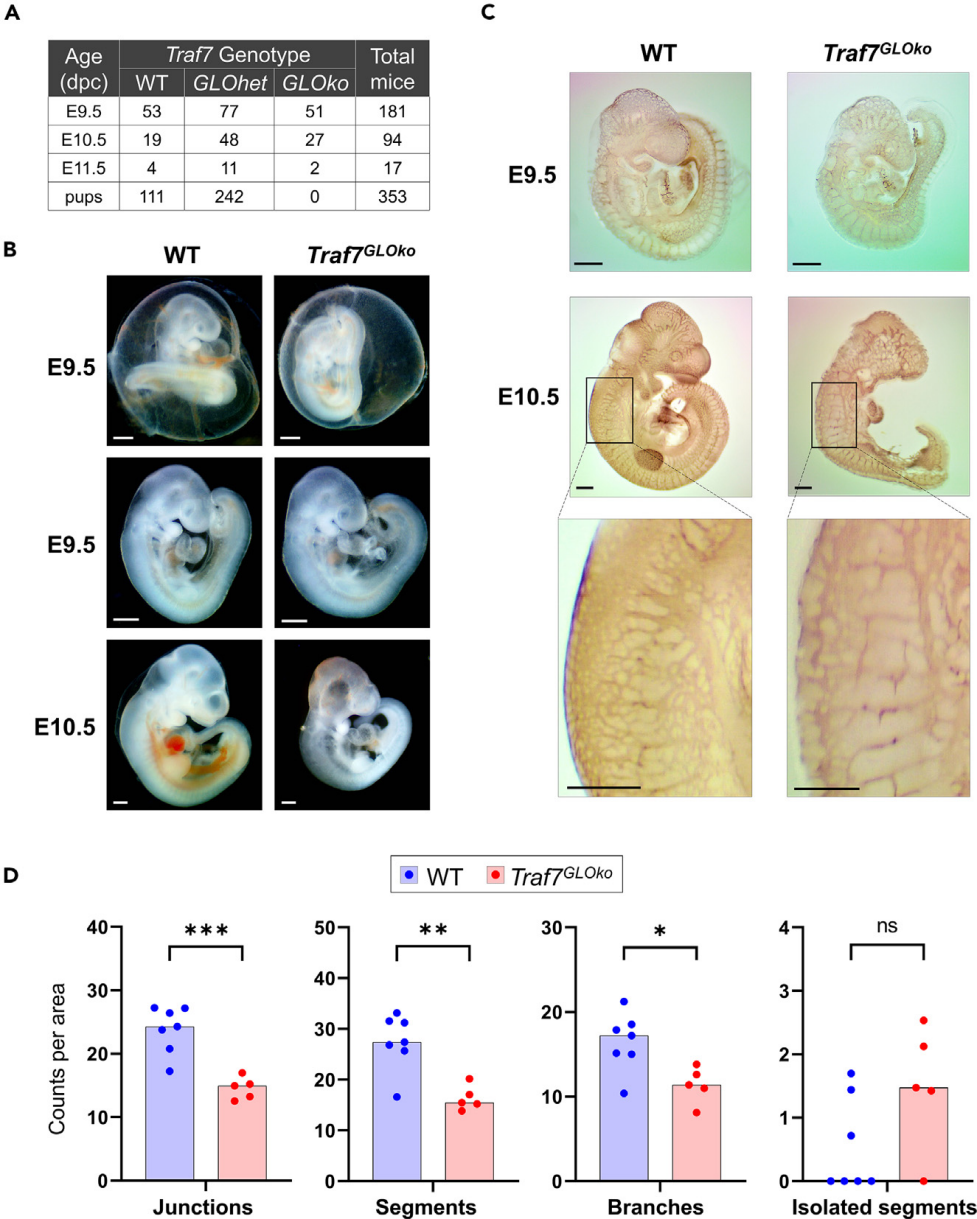
Targeted deletion of *Traf7* gene causes embryonic lethality (A) Genotyping analysis of offspring from mating between *Traf7*^*fl/fl*^*;E2a-Cre*^*+*^ (*Traf7*^*GLOhet*^) mice. *Traf7*^*GLOko*^ mice were never born, but embryonic genotypes (WT and *GLOhet*) showed the expected Mendelian distribution at E9.5 and E10.5. (dpc): days post-coitus. (B) Morphological analysis of littermate E9.5 WT and *Traf7*^*GLOko*^ embryos in lateral views inside the intact yolk sac (top panels) and without it (middle panels). Comparison of littermate E10.5 WT and *Traf7*^*GLOko*^ embryos (bottom panel). White scale bars are 0.5 mm. (C) Analysis of vascular structures in whole mount embryos. Littermate WT and *Traf7*^*GLOko*^ embryos at E9.5 and E10.5 were fixed and immuno-stained with an anti-PECAM-1 mAb (brown). Magnified insets show details of intersomitic vasculature. Black scale bars are 0.5 mm. (D) Computation of intersomitic vasculature in E10.5 *Traf7*^*GLOko*^ embryos. Each closed symbol represents an individual embryo. Bars show the Median. Asterisks indicate significant difference: (∗) p < 0.05; (∗∗) p < 0.005; (∗∗∗) p < 0.0005; (ns) not significant.

To investigate the function of TRAF7 *in vivo*, we generated mice with a conditionally targeted *Traf7* allele. The conditional allele contains *loxP* sites flanking exons 2 and 14, which include coding sequence for the first 373 amino acids of the protein through the middle of the first WD40 repeat (Figure S1A). Mice homozygous for the *Traf7*-floxed allele (*Traf*^*fl/fl*^) were healthy and fertile. To produce whole-body, global deletion of *Traf7* mice (*Traf7*^*GLOko*^ = global knockout), we crossed *Traf7-flox* mice with *E2a-Cre*-trangenic animals, which carry a *Cre* transgene under the control of the adenovirus EIIa promoter that targets expression of Cre recombinase ubiquitously to cells of the early mouse embryo.^29^ Heterozygous (*Traf7*^*GLOhet*^) mice were phenotypically normal and fertile. Genotyping of *Traf7*^*GLOhet*^ intercrosses revealed that no *Traf7*^*GLOko*^ pups were born (Figure 1A). The statistical analysis was performed to examine if there is deviation from expected Mendelian inheritance outcomes of the alleles during embryogenesis. The relation between these variables was not statistically significant for embryos on days E9.5 (*X*^2^ (2, *N* = 181) = 1.98, p = 0.3716) and E10.5 (*X*^2^ (2, *N* = 94) = 1.66, p = 0.4360). The deviation of *Traf7*^*GLOhet*^ progeny from Mendelian distribution of alleles was statistically significant for embryos at E11.5 (*X*^2^ (2, *N* = 17) = 10.72, p = 0.0047) and very statistically significant for newborns (*X*^2^ (2, *N* = 352) = 33.44, p = 0.0001). Therefore, analysis of timed mating revealed normal Mendelian segregation ratios for the disrupted *Traf7* alleles until embryonic day 10.5 (E10.5).

All E9.5 *Traf7*^*GLOko*^ embryos were viable, as determined by the presence of visible heartbeat (Videos S1, S2, S3, S4, and, S5). The *Traf7*^*GLOko*^ embryos and yolk sacs displayed normal appearance and no differences from wild-type (WT) littermates (Figure 1B, top and middle panel). After approximately E10, the mutant embryos displayed developmental delay and appeared smaller and paler than WT embryos; mutants also showed evidence of cerebral hemorrhage by E10.5 (Figure 1B, bottom panel). This phase in embryonic development coincides with the onset of embryonic blood flow and the establishment of a fully functional circulation,^30^ suggesting that TRAF7 may be important for the integrity of blood vessels in developing embryos. Because the phenotype of *Traf7*^*GLOko*^ embryos was similar to phenotypes observed in *Mekk3-*, *Mek5-* and *Erk5*-deficient embryos, which likewise died *in utero* around E10,^31^^,^^32^^,^^33^^,^^34^^,^^35^ we examined the expression of these proteins in *Traf7*^*GLOko*^ embryos. As shown in Figure S1D, there was no expression of TRAF7 in E9.5 *Traf7*^*GLOko*^ embryos, while expression of MEKK3, MEK5, and ERK5 remained normal, indicating that the expression levels of MEKK3, MEK5, and ERK5 were not altered by removal of TRAF7, and the lethality of *Traf7*^*GLOko*^ embryos was indeed associated with complete TRAF7 deficiency.

Video S1. Movie of heartbeat of E9.5 WT embryo #1, related to Figure 1B

Video S2. Movie of heartbeat of E9.5 WT embryo #2, related to Figure 1B

Video S3. Movie of heartbeat of *Traf7*^*GLOko*^ embryo #1, related to Figure 1B

Video S4. Movie of heartbeat of *Traf7*^*GLOko*^ embryo #2, related to Figure 1B

Video S5. Movie of heartbeat of *Traf7*^*GLOko*^ embryo #3, related to Figure 1B

Because the MEKK3-MEK5-ERK5 signaling cascade is essential for embryonic vascular integrity and cardiovascular development,^36^^,^^37^ we next investigated the expression of the endothelial cell marker platelet endothelial cell adhesion molecule 1 (PECAM-1, also known as CD31), in *Traf7*^*GLOko*^ embryos. There were no obvious visual differences in the appearance of blood vessel networks between WT and *Traf7*^*GLOko*^ embryos at E9.5 (Figure 1C, top panel). At E10.5, the blood vessels of *Traf7*^*GLOko*^ embryos appeared discontinuous and less branched compared to blood vessels in WT embryos (Figure 1C, middle panel). Quantification of intersomitic vessel structure using Angiogenesis Analyzer for ImageJ, which was developed for the analysis of *in vitro* angiogenesis patterns^38^ and later adopted for *in vivo* vascular changes in retina,^39^ demonstrated that blood vessels of E10.5 *Traf7*^*GLOko*^ embryos were significantly more fragmented compared to WT controls. There was lower numbers of junctions, segments, and branches per square unit. Consistently, the number of isolated segments was higher, although not significantly, in *Traf7*^*GLOko*^ embryos (Figure 1D). Thus, the initial vascularization occurs normally in *Traf7*^*GLOko*^ embryos, but the subsequent structural integrity of blood vessels was compromised, similarly to *Mekk3-*, *Mek5-* and *Erk5*-deficient embryos.^31^^,^^32^^,^^33^^,^^34^^,^^35^ These findings imply that TRAF7 may contribute to MEKK3-MEK5-ERK5 signaling pathway during embryonic development.

### *Traf7*-deficient embryos display abnormal heart development

**Figure 2:**
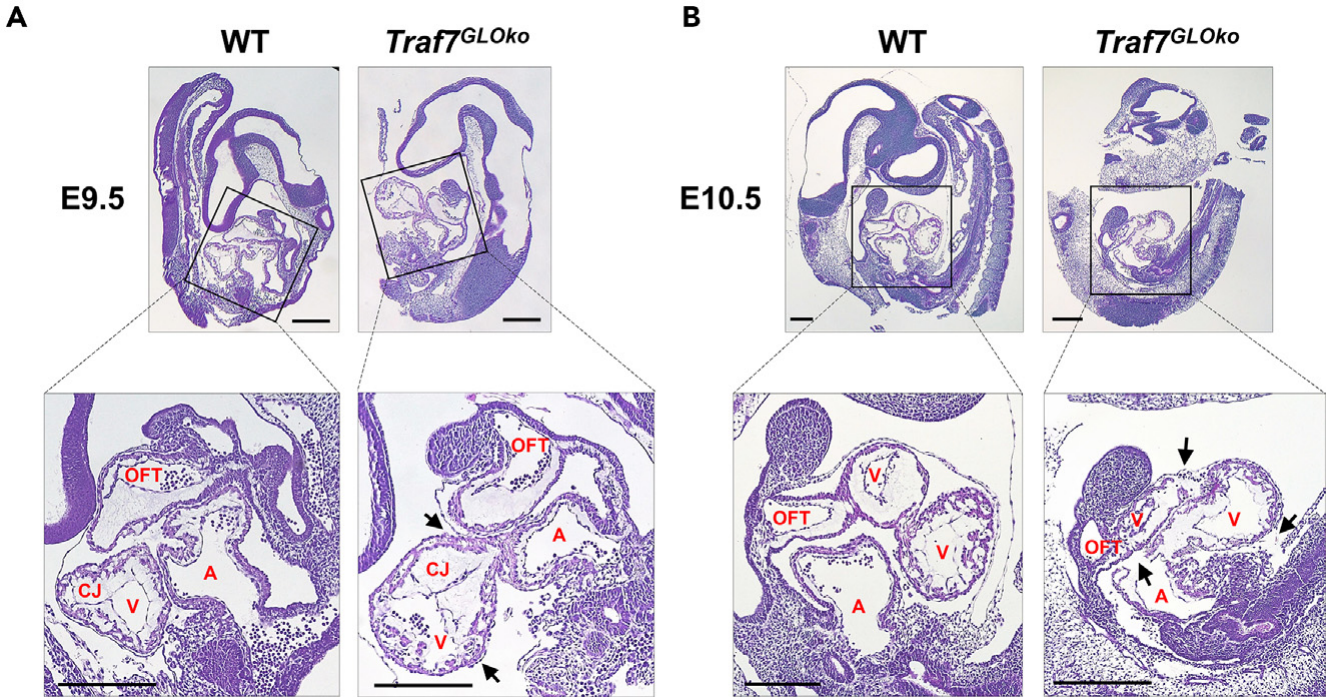
*Traf7* deletion caused defects in heart development (A and B) Hematoxylin and eosin staining of sagittal sections of E9.5 (A) and E10.5 (B) embryos. Pulmonary outflow tract (OFT), atrium (A), ventricle (V), cardiac jelly (CJ). Black scale bars are 0.5 mm. Damaged tissues in *Traf7*^*GLOko*^ embryos are indicated by arrows.

Although, histological analysis of E9.5 embryos revealed that *Traf7*^*GLOko*^ embryos had normal morphology (Figure 2A), the pericardium was broken in all *Traf7*^*GLOko*^ embryos we examined, probably due to lack of elasticity or excessive effusion. It is also possible that disruption of the pericardium happened during embryo handling, but we did not encounter a single broken pericardium in any of our WT embryos. Hence, E9.5 *Traf7*^*GLOko*^ hearts appeared to have a significantly different shape and protruded further than WT hearts (Figure 2A). A closer examination demonstrated grossly normal heart looping in *Traf7*^*GLOko*^ embryos with the atrioventricular canal extending more forward compared to WT controls. E9.5 *Traf7*^*GLOko*^ embryos also displayed an abnormal ventricle position accompanied by a higher deposition of cardiac jelly in endocardial cushions compared to WT counterparts. This phenotype is consistent with impaired MEKK3 signaling and low endocardium expression of KLF2, which was shown to control cardiac jelly production.^20^^,^^40^

At E10.5, *Traf7*^*GLOko*^ embryos displayed signs of tissue resorption and appeared more fragile than WT controls (Figure 2B). In contrast to WT hearts, which grew in size and developed interventricular foramen dividing the two ventricles, E10.5 *Traf7*^*GLOko*^ hearts did not increase in size and showed significant shrinkage of heart chambers with manifestations of degradation in surrounding tissue. Moreover, all experimental E10.5 *Traf7*^*GLOko*^ embryos without exception had broken heart walls and no blood cells in the heart cavities compared to their WT counterparts, indicating irreversible vascular failure by E10.5.

### Altered gene expression associated with TRAF7 deficiency in mouse embryo

**Figure 3:**
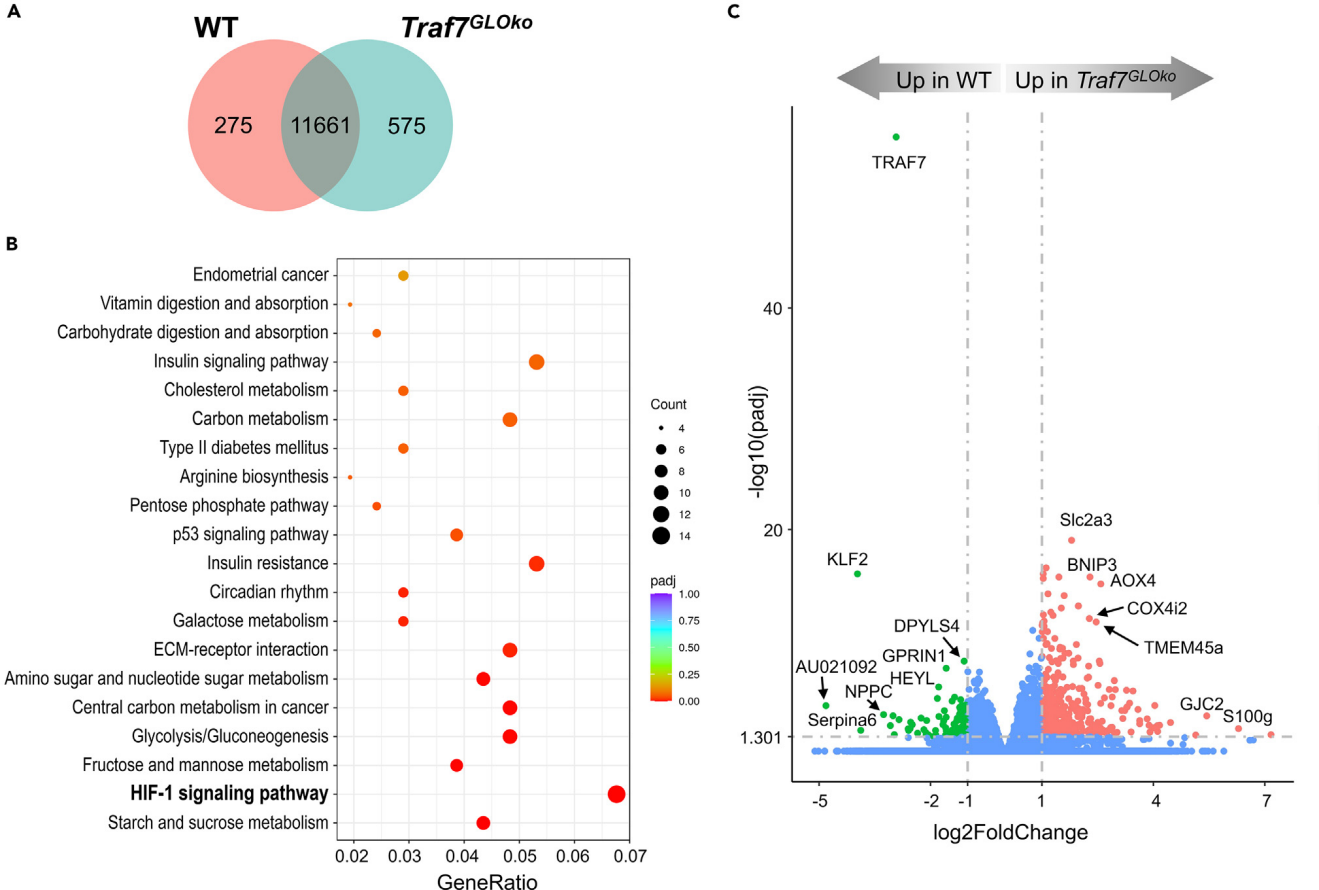
Altered gene expression associates with TRAF7 deficiency in mouse E9.5 embryos (A) Venn diagram showing the overlap of DEGs in WT and *Traf7*^*GLOko*^ mouse embryos by RNA-seq analysis. Significant DEGs had both an absolute log2 fold change ≥1 and an FDR adjusted p value ≤0.05. (B) Bubble plot of KEGG enrichment analysis of signaling pathways expressed in *Traf7*^*GLOko*^ versus WT embryos. Each bubble represents a KEGG pathway. Gene ratio (x axis) is the proportion of the total genes in a given pathway that is upregulated in the indicated group. (C) Volcano plot of RNA-seq analysis visualizing significant DEGs in WT versus *Traf7*^*GLOko*^ embryos: magnitude of change (*x axis*) vs. statistically significant p values (*y* axis). Points with p value less than 0.05 or fold change less than 2 (log_2_ = 1) are shown in blue.

To understand gene expression differences between E9.5 WT and *Traf7*^*GLOko*^ embryos, we compared their transcriptome profiles by RNA sequencing (RNA-seq). The analysis revealed that six WT embryos formed a tight cluster following principal-component analysis (PCA) (Figure S2A). Although six *Traf7*^*GLOko*^ embryos distributed more widely, three of them were in close proximity to the WT cluster, suggesting a recent branching of WT and *Traf7*^*GLOko*^ transcriptional profiles. Indeed, the majority of identified genes (11,661) were common between these two groups (Figure 3A), and only 275 and 575 of differentially expressed genes (DEGs) were specifically expressed in either WT or *Traf7*^*GLOko*^ embryos, respectively. Significant DEGs were defined as those that had both an absolute log2 fold change ≥1 and a false discovery rate (FDR)-adjusted p value ≤0.05 for each comparison independently. Hierarchical clustering based on gene expression profiles revealed high similarity of expression patterns between samples within each group and substantial differences between them (Figure S2B).

Gene set enrichment analysis in Kyoto Encyclopedia of Genes and Genomes (KEGG) pathway database demonstrated that WT and *Traf7*^*GLOko*^ embryos were different in the expression of many gene targets associated with the hypoxia-inducible factor 1 (HIF-1) signaling pathway (Figure 3B), suggesting embryonic distress resulting from insufficient blood flow in the absence of TRAF7. Detailed analysis of specific genes on a volcano plot demonstrated that *Traf7*^*GLOko*^ embryos were missing *Traf7*, as expected (Figure 3C). The most prominent downregulated DEG was the zinc-finger transcription factor *Klf2*, a key flow-responsive gene in endothelial cells.^41^ Interestingly, WT embryos also expressed approximately 1.5-fold higher levels of the *Klf2* homolog *Klf4* than *Traf7*^*GLOko*^ embryos, but without statistical significance (Table S1). Next, we validated the downregulated expression of *Klf2* in *Traf7*^*GLOko*^ embryos by quantitative RT-PCR (Figure S2C). Importantly, the expression of *Cd31* was the same in both types of embryos, suggesting a comparable number of WT and *Traf7*^*GLOko*^ endothelial cells. Because *Klf2* is almost exclusively expressed in the endothelium during mouse embryonic development, and because it was shown to be selectively induced by fluid shear forces^26^^,^^27^^,^^42^ or biomechanical stimuli via the MEK5-ERK5 pathway,^28^^,^^42^ our findings suggest an impaired MEKK3-MEK5-ERK5-KLF2 signaling cascade in *Traf7*^*GLOko*^ embryos.

The DEG with the highest level of expression in WT embryos compared to *Traf7*^*GLOko*^ embryos was *AU021092* (Figure 3C), a gene of unknown function. Because this gene is expressed by cardiomyocytes in E8.5 embryos,^43^ these results imply an abnormal heart function in *Traf7*^*GLOko*^ embryos. The other two DEGs downregulated in *Traf7*^*GLOko*^ embryos were *Serpina6*, also known as transcortin, and *Nppc*, also known as C-type natriuretic peptide (CNP). While transcortin is a major corticosteroid transporter in the blood, NPPC is a major heart-protective natriuretic peptide upregulated by shear stress in endothelial cells.^44^^,^^45^ One of the genes that was increased in *Traf7*^*GLOko*^ embryos, compared to WT counterparts, was *BCL2/adenovirus E1B interacting protein 3* (*Bnip3*), a pro-apoptotic gene induced by hypoxia.^46^^,^^47^^,^^48^ Interestingly, *Erk5*^*−/−*^ embryos also displayed low expression of *Klf2* and high expression of *Bnip3* when compared to WT controls.^25^ Taken together, these results once again suggest that TRAF7 is an integral part of vascular integrity regulated by MEKK3-MEK5-ERK5-KLF2 pathway in embryonic development.

### Endothelial-specific deletion of *Traf7* confers embryonic lethality

**Figure 4:**
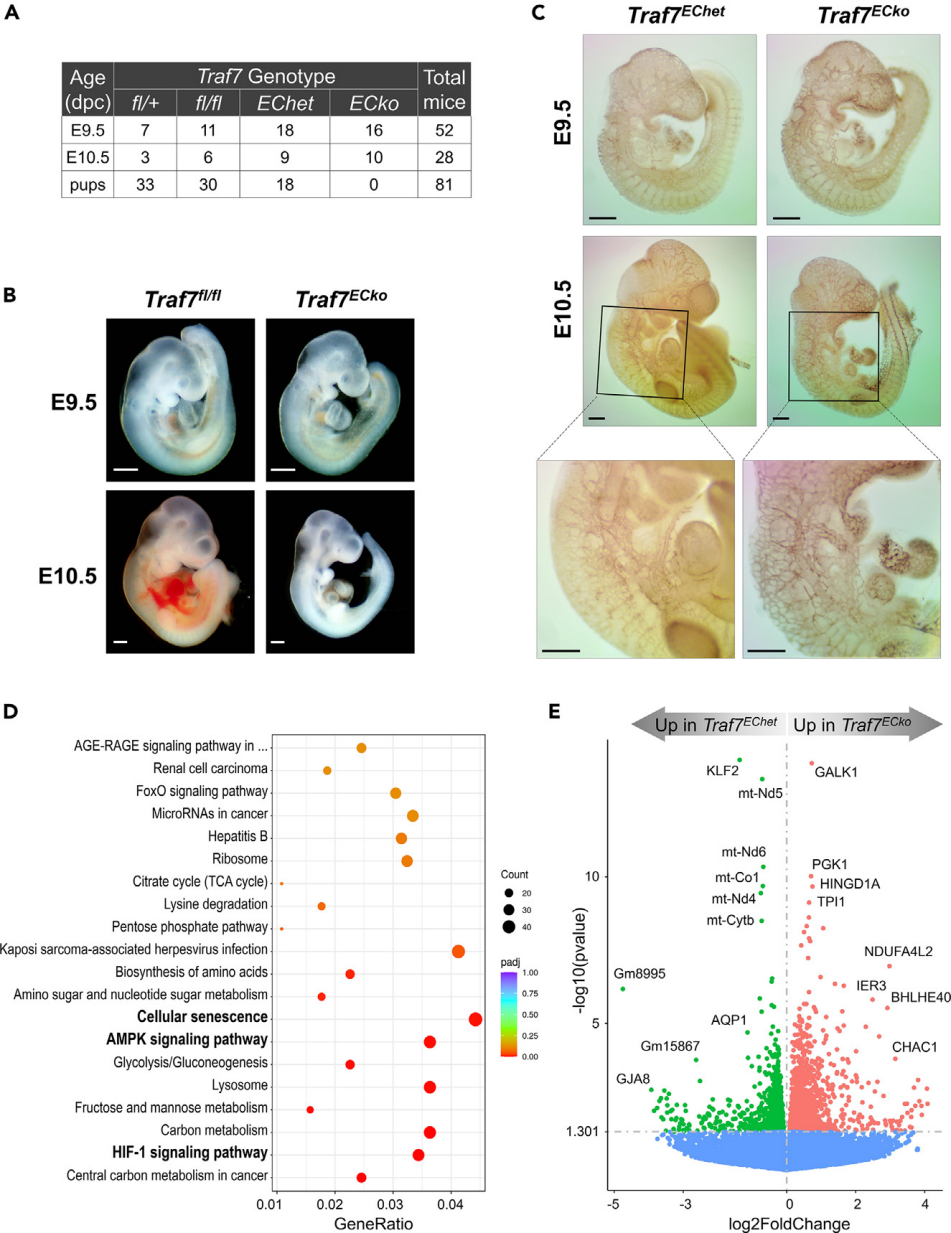
Endothelial-specific deletion of *Traf7* confers embryonic lethality and altered gene expression associates with endothelium-specific TRAF7 deficiency (A) Genotyping analysis of progeny following mating *Traf7*^*fl/fl*^ mice with *Traf7*^*fl/+*^*;Tie2-Cre*^*+*^ mice. *Traf7*^*fl/fl*^*;Tie2-Cre*^*+*^ (*Traf7*^*ECko*^) pups were never born, but genotyping of E9.5 and E10.5 embryos demonstrated the expected Mendelian distribution. (dpc): days postcoitum. (B) Morphological analysis of littermate control (*Traf7*^*fl/fl*^) and *Traf7*^*ECko*^ embryos at E9.5 and E10.5 in lateral views. White scale bars are 0.5 mm. (C) Visual analysis of embryonic vascular structures. Littermate control (*Traf7*^*EChet*^*)* and *Traf7*^*ECko*^ embryos at E9.5 (top panels) and E10.5 (middle panels) were fixed and immuno-stained with an anti-PECAM-1 mAb. Heart regions from E10.5 (middle panels) are shown at higher magnification in the bottom panels. Black scale bars are 0.5 mm. (D) Bubble plot of KEGG enrichment analysis of signaling pathways downregulated in *Traf7*^*ECko*^ versus *Traf7*^*EChet*^ (control) embryos at E9.5. Each bubble represents a KEGG pathway. Gene ratio (x axis) is the proportion of the total genes in a given pathway that is upregulated in the indicated group. (E) Volcano plot of RNA-seq analysis visualizing significant DEGs in *Traf7*^*ECko*^ versus *Traf7*^*EChet*^ (control) embryos at E9.5. Magnitude of change (*x*-axis) vs. statistically significant p values (*y* axis). Points with p value less than 0.05 are shown in blue.

To examine whether endothelium-specific expression of TRAF7 is required during embryonic development, we crossed *Traf7-*floxed mice with *Tie2-Cre*^*+*^ animals.^49^ The *Tie2-Cre* transgene drives *Cre* expression throughout early endothelium and in hematopoietic cells, but not in smooth or cardiac muscle.^50^ The intercross produced no *Traf7*^*fl/fl*^*;Tie2-Cre*^*+*^ pups (*Traf7*^*ECko*^ = endothelial cell knockout), indicating that conditional deletion of *Traf7* in endothelial cells leads to embryonic lethality (Figure 4A). Analysis of timed mating revealed ratios of progeny were non-Mendelian; specifically, the observed proportion of *Traf7*^*ECko*^ progeny (0%) differed from expected 25% (*Χ*^2^ (3, *N* = 81) = 27, p < 0.0001). Interestingly, the proportion of *Traf7*^*EChet*^ newborn pups (22.2%) was also lower than the expected 33% of remaining pups after *Traf7*^*ECko*^ embryonic lethality; *Χ*^2^ (2, *N* = 81) = 5, p = 0.03), suggesting that a single copy of *Traf7* in some embryos was not sufficient for survival during gestation. As with *Traf7*^*GLOko*^ embryos, E9.5 *Traf7*^*ECko*^ embryos were indistinguishable from *Traf7*^*fl/fl*^ (control) littermates, while E10.5 *Traf7*^*ECko*^ embryos appeared to be smaller and underdeveloped compared to controls (Figure 4B). In addition, some *Traf7*^*ECko*^ embryos were bloodless and/or had visible hemorrhages. Vascular development of E9.5 embryos was assessed by staining with anti-CD31 antibody and looked intact in both *Traf7*^*EChet*^ and *Traf7*^*ECko*^ embryos (Figure 4C). In contrast, E10.5 *Traf7*^*ECko*^ embryos displayed fragile blood vessels with interrupted connections in the head and in the back of the trunk where intersomitic arteries fuse to dorsal longitudinal anastomotical vessels. Quantification of intersomitic vessel structure demonstrated that blood vessels of E10.5 *Traf7*^*ECk*o^ embryos were significantly more fragmented compared to *Traf7*^*EChet*^ controls. There were significantly lower numbers of junctions, segments, and branches, while higher numbers of isolated segments in*Traf7*^*GLOko*^ embryos (Figure S3). Overall, it appears that *Traf7*^*ECko*^ embryos die around E10, which is similar to the timing of death of *Traf7*^*GLOko*^ embryos. Taken together, these results indicate that TRAF7 is essential for normal endothelium function during embryonic development.

To gain insight into the genes regulated by TRAF7 in embryonic endothelial cells, we performed RNA-seq analysis of *Traf7*^*ECko*^ embryos at E9.5. *Traf7*^*EChet*^ embryos were chosen as a control group to cancel out any interference resulting from the presence of the *Tie2-Cre* transgene. Thus, both of these groups of embryos only differ by a single copy of *Traf7* in any given cell after *Cre*-dependent deletion. The analysis revealed that *Traf7*^*EChet*^ embryos formed a relatively tight cluster following PCA (Figure S4A), while *Traf7*^*ECko*^ embryos failed to form a compact cluster. Three *Traf7*^*ECko*^ embryos were in close proximity to the control cluster, suggesting incomplete deviation of the two transcriptional profiles, probably due to inefficient deletion of the floxed *Traf7* gene. Indeed, the vast majority of identified genes (11,787) were common between the two groups, and only 172 and 218 of the DEGs were upregulated in control and *Traf7*^*ECko*^ embryos, respectively (Figure S4B). Nevertheless, hierarchical clustering of transcriptome profiles revealed high sample similarity within each group and a good separation between groups (Figure S4C). Gene set enrichment analysis in the KEGG pathway database unveiled enrichment of cellular senescence, AMP-activated protein kinase (AMPK), and HIF-1 signaling in *Traf7*^*ECko*^ embryos compared to *Traf7*^*EChet*^ (Figure 4D), suggesting a deviation in energy balance between the two groups due to *Traf7* deficiency.

In agreement with transcriptome analysis of *Traf7*^*ECko*^ embryos, *Klf2* turned up to be the most statistically significant DEG with lower expression in embryos with endothelial-specific *Traf7* deletion compared to the controls (Figure 4E and Table S2). Downregulation of *Klf2* in *Traf7*^*ECko*^ embryos was confirmed with quantitative RT-PCR (Figure S4D). There was also decreased expression of the nitrogen oxide-transporting transmembrane pore aquaporin-1 (*Aqp1*) previously identified as a direct subject to KLF2-mediated positive regulation in endothelial cells,^51^ suggesting dysregulated KLF2 activity and impaired endothelial cell function in *Traf7*^*ECko*^ embryos. Out of broadly expressed genes, *Traf7*^*ECko*^ embryos demonstrated lower expression of *mt-Nd6*, *mt-Co1*, *mt-Nd4*, and *mt-CytB*, subunits of the mitochondrial membrane respiratory chain enzymes, indicating attenuated aerobic respiration in tissues of *Traf7*^*ECko*^ embryos. Consistently, galactokinase *Galk1* and mitochondrial *Ndufa4l2*, two direct targets of HIF-1,^52^^,^^53^ were among the most statistically significant DEGs highly expressed in *Traf7*^*ECko*^ embryos compared to *Traf7*^*EChet*^ counterparts (Figure 4E). These results are in agreement with recent observation that the mitochondrial pathway participates in the expression of KLF2-dependent genes and improves vascular remodeling.^54^

*Traf7*^*ECko*^ embryos also displayed higher expression of immediate-early response genes, *Ier3* and basic-helix-loop-helix transcription factor *Bhlhe40*, indicating once again embryonic distress resulting from insufficient oxidation in the absence of TRAF7. On the other hand, *Klf4* was not among DEGs significantly upregulated in *Traf7*^*ECko*^ embryos compared to *Traf7*^*EChet*^ controls (Table S2), implying that the transcription of *Klf4* is not regulated by TRAF7 in embryonic endothelial cells.

### Conditional deletion of *Traf7* in postnatal endothelium leads to brain hemorrhage

**Figure 5:**
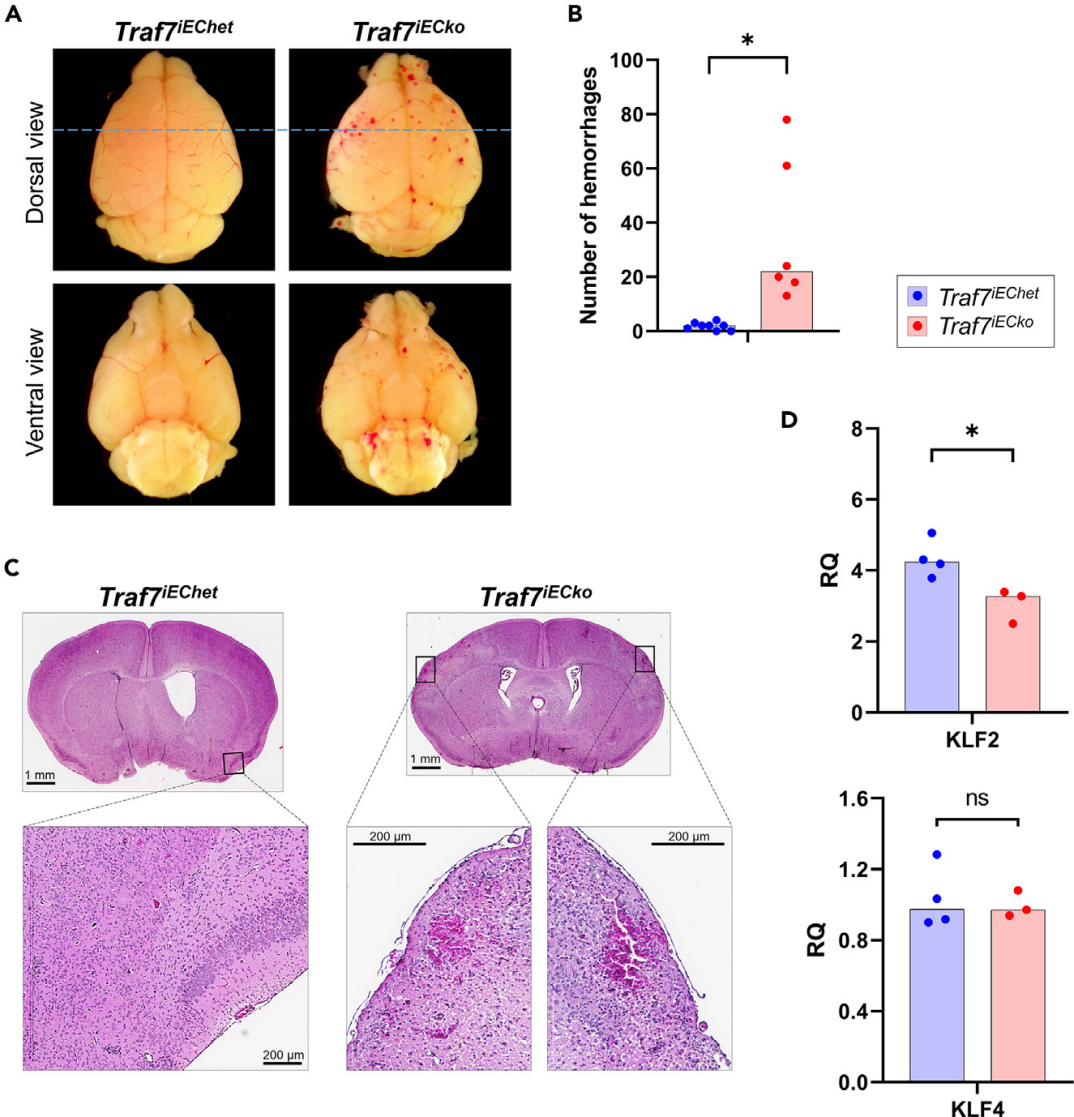
Conditional deletion of *Traf7* in postnatal endothelium leads to brain hemorrhage (A) Dorsal and ventral views of representative embryonic brains from P13 *Traf7*^*iEChet*^ and *Traf7*^*iECko*^ pups collected after daily tamoxifen administration. (B) Quantification of brain hemorrhagic foci in *Traf7*^*EChet*^ (n = 8) and *Traf7*^*ECko*^ (n = 8) pups. (C) H&E-stained coronal brain sections at the position indicated by the dotted blue line in (A). (D) mRNA expression analysis of *Klf2* and *Klf4* in brains of P7 *Traf7*^*iEChet*^ versus *Traf7*^*iECko*^ pups. Each closed symbol represents an individual embryo. Bars show the Median. Asterisks indicate significant difference: (∗) p < 0.05; (ns) not significant.

Since global or endothelial-specific deletion of *Traf7* resulted in embryonic lethality, we next examined whether TRAF7 expression is required for the integrity of postnatal endothelium. We crossed *Traf7*^*fl/fl*^ mice to *Cdh5(PAC)-Cre*^*ERT2*^*-*trangenic animals, which demonstrate high levels of recombinase-mediated gene deletion in vascular endothelial cells following administration of tamoxifen.^55^^,^^56^ *Traf7* deletion was induced starting at postnatal day 0 (P0) by daily oral feeding of tamoxifen. Twelve days after the initiation of tamoxifen administration, we noted that one of *TRAF7*^*fl/-*^*·Cdh5(PAC)-CreERT2* (*Traf7*^*iECko*^ = inducible endothelial cell knockout) mice developed a pronounced ataxia with seizure-like behavior. At this point, we euthanized all experimental mice in this group and examined the brains. The gross inspection of whole brains demonstrated that, in contrast to *Traf7*^*iEChet*^ littermates, *Traf7*^*iECko*^ mice exhibited prominent focal hemorrhages (Figure 5A), while the quantification revealed a significantly higher number of hemorrhagic foci in *Traf7*^*iECko*^ brains compared to the controls (Figure 5B). A closer examination of neonatal brains confirmed bleeding in *Traf7*^*iECko*^ mice (Figure 5C). Blood vessels in the brain and choroid plexus of the mutant animals were prone to rupture, releasing erythrocytes. The brain was the only affected organ in our experiments, probably due to extensive vascular remodeling of the brain during postnatal development.^57^ The analysis of *Klf2* and *Klf4* in P7 brains revealed a significantly lower expression of *Klf2*, but not of *Klf4*, in *Traf7*^*iECko*^ brains compared to *Traf7*^*iEChet*^ littermates, mirroring the results obtained in *Traf7*-deficient embryos (Figure 5D). Our finding that postnatal endothelial-specific deletion of *Traf7* resulted in brain hemorrhage is in agreement with previously published results demonstrating that *Mekk3* is required for the integrity of postnatal vasculature.^58^

### Association of TRAF7 with SCRIB

**Figure 6:**
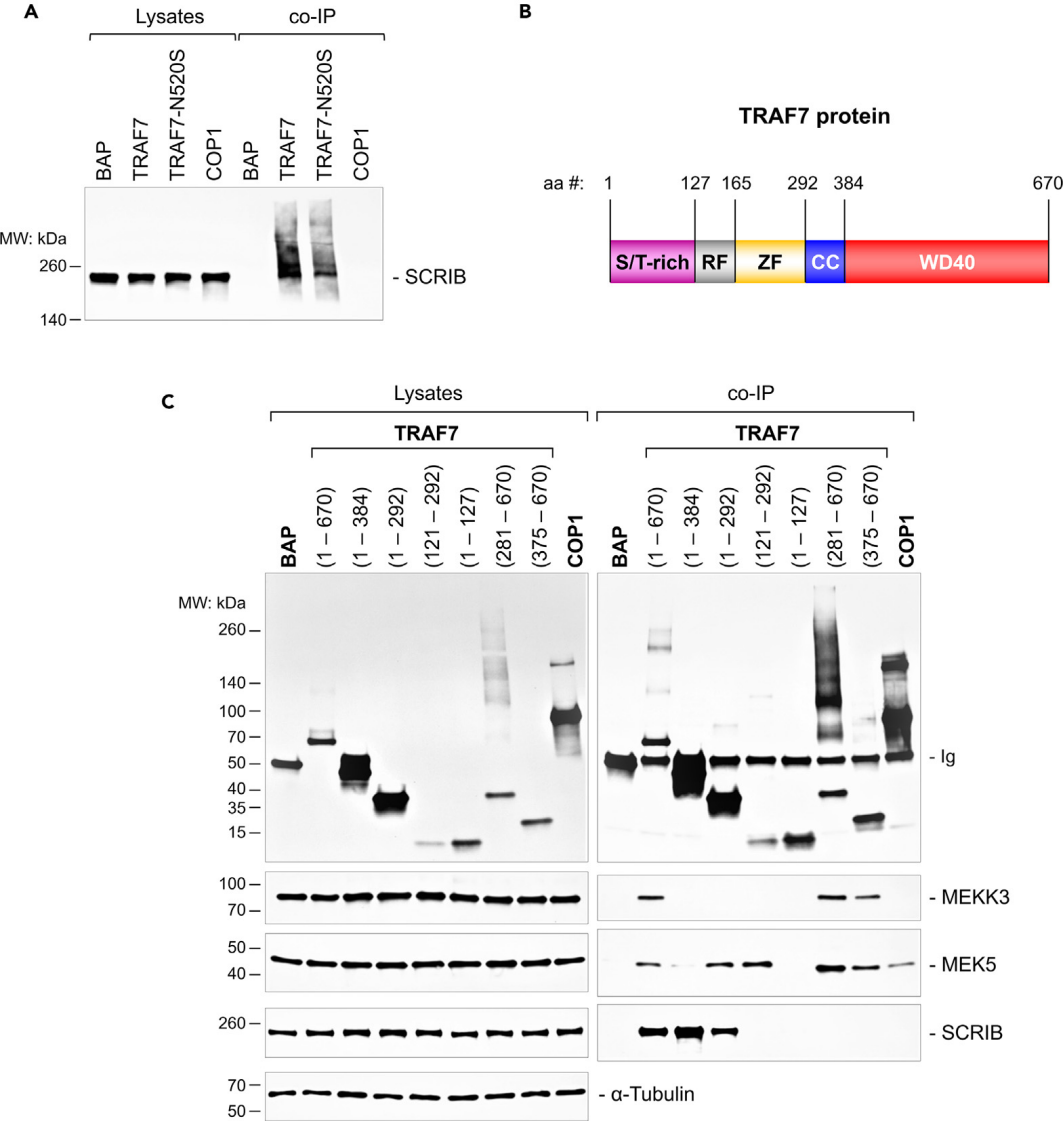
Association of TRAF7 with SCRIB (A) Western blot analysis of SCRIB protein expression and its co-immunoprecipitation (co-IP) with TRAF7 using HEK293 whole-cell lysates and indicated antibodies. BAP (bacterial alkaline phosphatase), COP1, TRAF7, or TRAF7^N520S^ were overexpressed as FLAG-fusion proteins, and co-IP was done with anti-FLAG antibody. Co-IP lanes show amount of protein in 10 times lysate input volume. (B) Schematic structure of TRAF7 protein. S/T-rich (serine/threonine-rich), RF (ring finger), ZF (Zinc finger), CC (coiled coil), and WD40 domains are depicted by different colors. Amino acids numbers (aa #) above the diagram correspond to border amino acids in truncated FLAG-tagged TRAF7 proteins used in this study. (C) Western blot analysis of MEKK3, MEK5, and SCRIB protein expression and their co-immunoprecipitation (co-IP) with overexpressed truncated FLAG-TRAF7 proteins using HEK293 whole-cell lysates and indicated antibodies. Co-IP was done with anti-FLAG antibody. Anti-α-Tubulin antibody was used as the loading control. Co-IP lanes show amount of protein in 10 times lysate input volume. Blots shown in panels (A) and (C) are representative of at least four independent experiments.

Although the role of the MEKK3-MEK5-ERK5-KLF2 pathway in blood flow-induced shear stress signaling in endothelial cells has been extensively studied over the years,^36^^,^^37^^,^^59^ the identity of the upstream regulator/s of this signaling cascade is still obscure. To discover additional TRAF7-interacting molecules, we used TRAF7 to pull down associated proteins. In order to do this, the coding regions of full-length TRAF7 and meningioma mutant TRAF7^N520S^ were expressed as fusion proteins with FLAG epitope tags at the N terminus. Plasmids encoding FLAG-fusion proteins of bacterial alkaline phosphatase (BAP) and COP1 were used as negative controls. We transfected HEK293 cells with vectors expressing fusion proteins and precipitated associated proteins with anti-FLAG magnetic beads (Figure S5A). Interestingly, bead-bound TRAF7 and TRAF7^N520S^ formed high-molecular-weight complexes, which were partially resistant to solubilization in the reducing Laemmli buffer. Both TRAF7 fusion proteins associated with endogenous MEKK3, MEKK2, and MEK5, but not MARK2, another kinase that was also previously shown to interact with TRAF7.^12^ There also was no ERK5 in TRAF7 precipitates, indicating that ERK5 may not be a part of the TRAF7 complex. Importantly, MEKK2, MEKK3, and MEK5 precipitated with neither BAP nor COP1, indicating specificity of binding to TRAF7. Next, we performed mass spectrometry analysis of TRAF7-interacting proteins precipitated from HEK293 cells. After subtracting non-specific proteins precipitated with BAP beads, SCRIB (also known as SCRB1), a homolog of *Drosophila melanogaster* SCRIBBLE, appeared to be the most abundant of TRAF7-interacting proteins (Figure S5B). Western blot analysis revealed that SCRIB specifically precipitated with both TRAF7 and TRAF7^N520S^, but not with BAP or COP1 (Figure 6A). While coincidental association of TRAF7 with SCRIB was first mentioned in the original TRAF7-MEKK3 study, no results were shown.^12^ More recently, SCRIB was also found as one of 79 proteins associated with TRAF7, and TRAF7 was identified as one of 95 proteins associated with SCRIB in human interactome studies.^19^ Taken together, these results demonstrate that TRAF7 interacts with SCRIB in addition to MEKK2, MEKK3, and MEK5.

To further characterize the association of MEKK3, MEK5, and SCRIB with TRAF7, we expressed truncated TRAF7 fragments and co-precipitated endogenous associated proteins (Figures 6B and 6C). In agreement with previous reports,^12^^,^^14^ the C-terminal WD40 domain (375–670) was required and sufficient for interaction of TRAF7 with MEKK3. Interestingly, MEK5 associated with two distinct regions in TRAF7. The first TRAF7 region (121–292) comprises the RING finger and zinc-finger domains, and the second region (375–670) includes the WD40 domain, which also binds MEKK3. The shortest truncated mutant of TRAF7 able to bind SCRIB was TRAF7(1–292). Two shorter mutants, TRAF7(1–127) and TRAF7(121–292), did not associate with SCRIB, suggesting that a fragment consisting of the N-terminal Serine-Threonine (S/T)-rich RING finger and ZF of TRAF7 was necessary for interaction with SCRIB. Thus, there are two non-overlapping regions in TRAF7: the N-terminal region of TRAF7 that binds SCRIB and the C-terminal that binds MEKK3. Interestingly, MEK5 binds to both regions.

To examine whether TRAF7 directly interacts with SCRIB, we ectopically co-expressed both of them as fusion fluorescent proteins (Figure S6). Full-size SCRIB was expressed as a fusion with EGFP, while full-size TRAF7 and its fragments were expressed as fusion proteins with mCherry. When expressed alone, the majority of full-size TRAF7 was localized in a discrete vesicular pattern in the cytosol, as was previously shown.^12^ In contrast, TRAF7(1–292) was diffusely distributed throughout the cytoplasm, while TRAF7(1–384) formed even bigger aggregates in the cytoplasm, suggesting that the CC domain of TRAF7 is important for self-interaction. When expressed alone, SCRIB-EGFP showed a diffuse intracellular distribution, but when expressed together with truncated TRAF7 fragments, it joined the TRAF7-containing aggregates, suggesting direct interaction of two proteins.

### TRAF7 and SCRIB are essential for phosphorylation of ERK5 induced by fluid flow

**Figure 7:**
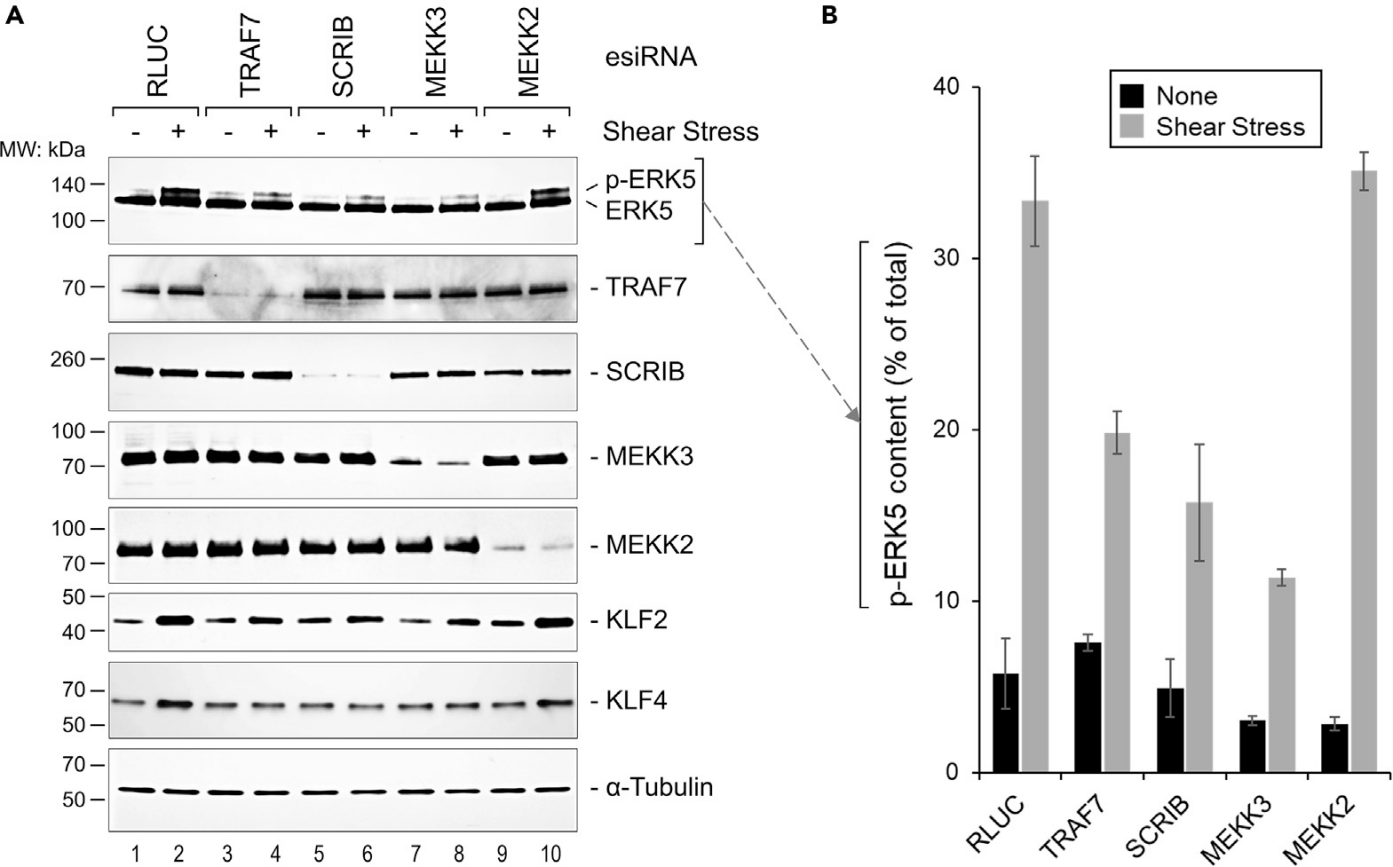
TRAF7 and SCRIB are essential for phosphorylation of ERK5 in endothelial cells exposed to shear stress (A) Western blot analysis of ERK5 phosphorylation (p-ERK5) and TRAF7, SCRIB, MEKK3, MEKK2, KLF2, and KLF4 protein expression in HUVECs under normal (−) or shear stress (+) conditions, with addition of esiRNA specifically inhibiting each of the proteins as indicated. Anti-*Renilla* luciferase (RLUC) esiRNA was used as a negative control. Anti-α-Tubulin antibody was used as the loading control. The results are representative of least three independent experiments. (B) Protein gel quantification analysis of the top western blot panel in (A). p-ERK5 protein levels were calculated as percent of total ERK5 protein content in corresponding lanes using GeneTools from Syngene software. Western blots from four independent experiments were analyzed. Data are presented as Mean ± StDev.

As a key integrator of fluid shear stress signaling, ERK5 is required for the maintenance of blood vessel integrity and vascular homeostasis.^31^^,^^32^^,^^34^^,^^60^ It is activated by fluid flow shear stress in endothelial cells following phosphorylation by MEK5.^61^ To understand whether TRAF7 and SCRIB are important for phosphorylation of ERK5 in endothelial cells activated by fluid flow, we exposed a confluent monolayer of human umbilical vein endothelial cells (HUVECs) to pulsating shear stress on an orbital shaker.^62^ Western blot analysis showed a notable upward shift in molecular weight of ERK5 in cells exposed to shear stress and transfected with negative control endoribonuclease-prepared small interfering RNAs (esiRNAs) against Renilla luciferase (RLUC) and MEKK2 (Figure 7, lanes 2 and 10). This shift was consistent with expected activation and phosphorylation of ERK5, which is not mediated by MEKK2.^61^^,^^63^ In contrast, silencing of TRAF7, SCRIB, and MEKK3 resulted in downregulated phosphorylation of ERK5 following exposure to shear stress, suggesting that TRAF7 and SCRIB together with MEKK3 may be important for phosphorylation of ERK5 induced by blood flow in endothelial cells. Because both KLF2 and KLF4 were shown to be downstream of increased MEKK3-MEK5-ERK5 signaling in cerebral cavernous malformations,^64^^,^^65^ we next examined the expression of KLF2 and KLF4 in shear stress-exposed HUVECs. The results demonstrated an upregulation of KLF2 as well as KLF4 proteins in HUVEC transfected with negative control esiRNAs against RLUC and MEKK2 after exposure to shear stress (lanes 2 and 10 in Figure 7A). In contrast, there was a lower upregulation of these transcription factors in cells transfected with esiRNAs against TRAF7, SCRIB, or MEKK3 (lanes 4, 6 and 8 in Figure 7A), indicating that TRAF7 as well as SCRIB are important for upregulation of KLF2 and KLF4 in HUVEC in response to shear stress *in vitro*.

## Discussion

Blood flow through vessels exerts circulatory pressure and fluid shear stress on the endothelial cells that line the luminal surface.^66^ The level of biomechanical stress varies from region to region within the vasculature and plays a critical role in shaping the vascular system to optimize perfusion. The blood flow-responsive endothelial transcription factor KLF2 is known to be activated by shear stress signaling mediated through the MEKK3-MEK5-ERK5 axis.^37^ Indeed, differential *Klf2* expression exhibited the highest statistical significance with substantial downregulation in global as well as endothelial-specific *Traf7*-deficient embryos compared to controls, suggesting that like MEKK3, MEK5, and ERK5, TRAF7 is necessary for *Klf2* expression in endothelial cells. It is interesting, however, that *Klf2*-deficient embryos die almost two days later than mice lacking *Traf7*, *Mekk3*, *Mek5*, or *Erk5*, e.g., between E11.5 to E14.5 depending on the background.^40^^,^^42^^,^^67^^,^^68^ Moreover, *Klf2*-deficient embryos die due to embryonic heart failure but not from anemia or structural vascular defects. These results suggest that genes other than *Klf2* may be the cause of early death of *Traf7*^*GLOko*^ embryos.^42^ One of those genes may be *Klf4* because double-knockout *Klf2*^−/−^;*KLF4*^−/−^ embryos die by E10.5 due to disruptions of the endothelial cell layer of the primary head vein.^69^ However, no significant difference was observed in the expression of *Klf4* in neither *Traf7*^*GLOko*^ nor *Traf7*^*ECko*^ embryos compared to their controls (Tables S1 and S2), indicating that other genes may also contribute to impaired endothelial integrity in *Traf7*^*GLOko*^ embryos.

Although the molecular mechanism of shear stress sensing in endothelial cells has been extensively studied, much still remains to be investigated due to the complexity of blood flow through the vascular network, including variations in intensity, direction, and pulsatility. Here, we demonstrate that in addition to MEKK3 and MEK5, TRAF7 binds to a planar cell polarity protein, SCRIB, which was originally identified in *Drosophila* as a large cytoplasmic protein containing 16 leucine-rich repeats (LRR), two leukemia-associated-protein (LAP)-specific domains, and four PSD95/Dlg1/zo-1 (PDZ) domains.^70^ In *Drosophila*, it associates with discs-large (DLG), and lethal-giant larvae (LGL) proteins, while in mammals SCRIB binds numerous partners and functions as a master scaffold in planar cell polarity, adhesion, synaptogenesis, and proliferation processes.^71^ In cultured mammalian epithelial cells, SCRIB localizes to the basolateral membrane and appears to overlap with adherens junctions.^72^ It plays important roles in cell adhesion, planar polarity, and asymmetric divisions.^73^

There are conflicting reports regarding the role of SCRIB in endothelial cells. Although the functional consequences of these interactions remain unknown, SCRIB was shown to bind non-filamentous vimentin near membrane borders between cells in confluent HUVEC cultures.^74^ In another study, SCRIB interacted with integrin α5 in HUVECs and was shown to be important for directed migration and angiogenesis of intersegmental vessels in zebrafish embryos.^75^ In mice, endothelial-specific deletion of *Scrib* significantly impairs LPS-induced vascular cell adhesion protein 1 (VCAM1) expression and leukocyte adhesion in response to TNF treatment, suggesting that SCRIB facilitates endothelial inflammatory signaling.^76^ On the other hand, deletion of *Scrib* in *ApoE*-deficient mice impairs vascular permeability and promotes atherosclerosis development, indicating that SCRIB contributes to anti-inflammatory responses in endothelial cells.^77^ The latter observation is consistent with the inward arterial remodeling and accelerated atherosclerosis seen in *Mekk3*-deficient mice.^78^ These results imply that SCRIB and MEKK3 may have an overlapping function, which supports our finding that SCRIB knockdown reduced shear stress-induced phosphorylation of ERK5 in HUVECs.

Human *Scrib* mutations are associated with the most severe type of neural tube closure defects, including craniorachischisis and spina bifida.^79^^,^^80^ Interestingly, the *circletail* mouse (*Scrib*^*Crc*^) closely mirrors the phenotypic defects described in humans.^81^^,^^82^ In *Scrib*^*Crc*^ mice, a spontaneous mutation due to a single base insertion after 3182 bp of coding sequence results in frameshift at codon 947 and truncation of SCRIB protein after the second PDZ domain, leaving intact exons for all LLRs, two LAP domains, and the first two PDZ domains. Nevertheless, *Scrib*^*Crc*^ embryos failed to initiate neural tube closure at E8.5 and exhibited an open neural tube from the midbrain/hindbrain boundary throughout the spine.^83^^,^^84^ Likewise, whole-body *Scrib*-knockout mice (*Scrib*^*Gko*^) die prenatally and display an array of developmental abnormalities, including neural tube closure defects and cochlear hair cell orientation defects.^85^ In these mice, the authors excised exons 4–12, encoding LLRs 4–16, but kept in place the coding sequence for LLRs 1–3, two LAP domains, and all PDZ domains. This may explain why the phenotype of *Scrib*^*Crc*^ *and Scrib*^*Gko*^ mice is very different from the phenotype of *Traf7*^*GLOko*^ mice, which had no obvious neural tube closure defects and died around E10 from failed blood vessel integrity. Thus, we cannot exclude that SCRIB might play a role in shear stress-dependent TRAF7-MEKK3-MEK5-ERK5 axis in endothelial cells during embryonic development because in both *Scrib*-knockout mice the majority of the SCRIB coding sequence was left intact in the genome. These leftover fragments might still bind TRAF7 and potentially play a role in TRAF7-dependent shear stress signaling in endothelial cells. Future studies are needed to assess whether SCRIB participates in the sensing of blood flow physical forces by the TRAF7-MEKK3-MEK5 complex during embryogenesis.

When TRAF7 was discovered as a MEKK3 synergistic partner in activation of NF-κB, p38, and c-June N-terminal kinase (JNK), it was demonstrated that TRAF7 is an E3 ubiquitin ligase capable of self-ubiquitination,^12^ although E3 ubiquitin ligase activity was not important for signal transduction in both original studies.^12^^,^^14^ Subsequently, TRAF7 was shown to ubiquitinate a number of molecules, including c-MYB,^16^ MYOD1,^17^ NEMO,^18^ FLICE-like inhibitory protein (c-FLIP_L_, also known as CFLAR),^86^ p53,^87^ and KLF4.^88^ In every study ubiquitination resulted in degradation of the substrate and activation of pro-inflammatory and/or pro-apoptotic pathways, such as NF-κB, or MAP kinases.^7^ For example, it was shown that TRAF7 associated with KLF4 through its ZF-CC region and was responsible for ubiquitination and proteasome-dependent degradation of KLF4 through its RING-dependent E3 ubiquitin ligase activity to promote hepatocellular carcinoma progression.^88^ In contrast to the above observations, we found that TRAF7 is a part of blood flow-induced shear stress-responsive MEKK3-MEK5-ERK5 signaling pathway, which plays an anti-inflammatory role in the endothelium.^37^ Moreover, we did not detect any significant differences in the expression of MEKK3 or MEK5 in *Traf7*^*GLOko*^ embryos compared to WT controls (Figure S1D). Furthermore, no significant changes in the expression of MEKK3, MEK5, or SCRIB were observed in the lysates of HEK293 cells ectopically expressing full size or fragments of TRAF7 (Figure 6C) or in TRAF7-deficient HUVECs exposed to shear stress (Figure 7). We also notice no increases in molecular weight of TRAF7-associated proteins, except SCRIB (Figure 6A), following association with TRAF7. The investigation whether higher-molecular-weight variants of SCRIB are the results of protein aggregation or ubiquitination would be an interesting subject of future investigations.

### Limitations of the study

In this study, we generated *Traf7*-deficient mice to elucidate the physiological role of TRAF7 and describe phenotypes resulted from global and endothelial cell-specific deletion of *Traf7*. Both *Traf7*^*GLOko*^- and *Traf7*^*ECko*^-deficient embryos failed to develop beyond day 10 of embryonic development due to impaired blood vessel integrity linking TRAF7 with MEKK3-MEK5-ERK5 signaling pathway. One possible mechanism through which TRAF7 modulates shear stress signaling is by association with SCRIB, a planar cell polarity protein. We show that SCRIB is important for shear stress-induced phosphorylation of ERK5 in HUVECs. Transfections of HUVECs with esiRNA worked consistently well, but plasmid transfections into HUVECs proved to be difficult and inefficient in our hands as it was shown before for HUVECs.^89^ This presented an obstacle for TRAF7 interaction studies in endothelial cells. Therefore, we performed all *in vitro* TRAF7 interaction studies in HEK293 cells. Major unanswered questions include how TRAF7 activates MEKK3-MEK5-ERK5 signaling, does it function as E3 ligase for possible ubiquitination of these associated proteins, as well as the molecular mechanism of endothelium weakness in TRAF7-deficient animals. These and other questions are a matter for future studies.

## STAR★Methods

### Key resources table

**Table:** 

REAGENT or RESOURCE	SOURCE	IDENTIFIER
**Antibodies**		

Monoclonal ANTI-FLAG® M2 antibody produced in mouse	Sigma-Aldrich	Cat#F1804; RRID: AB_262044
Immun-Star Goat Anti-Mouse (GAM)-HRP Conjugate	BIO-RAD	1705047; RRID:AB_11125753
Anti-CD31, rat monoclonal (MEC13.3) anti-mouse	Fisher Scientific	BDB557355
Goat anti-Rat IgG, polyclonal Affinity-Purified secondary Antibody, HRP	Fisher Scientific	OB3010-05
HA-Tag (C29F4) Rabbit mAb	Cell Signaling Technology	Cat#3724; RRID:AB_1549585
Anti-TRAF7 (WD40) antibody produced in rabbit	Sigma-Aldrich	Cat#HPA041229; RRID:AB_10794194
Goat anti-Rabbit IgG (H+L) Cross-Adsorbed Secondary Antibody, HRP (Invitrogen)	Fisher Scientific	Cat#31462; RRID:AB_228338
MEKK3 Rabbit anti-Human, Mouse, Polyclonal, Invitrogen	Fisher Scientific	Cat#PIPA578058
MEKK2, Rabbit polyclonal	Cell Signaling Technology	Cat#19607; RRID:AB_2798822
MEK5 (E4C4B) Rabbit monoclonal	Cell Signaling Technology	Cat#40737
SCRIB, Rabbit polyclonal	Cell Signaling Technology	Cat#4475; RRID:AB_10557101
Anti-KLF4, rabbit polyclonal	Sigma-Aldrich	Cat#SAB2107958
KLF2 Rabbit polyclonal, Invitrogen™	Fisher Scientific	Cat#PIPA540591
α-Tubulin (DM1A) Mouse monoclonal	Cell Signaling Technology	Cat#3873; RRID:AB_1904178
Anti-Sp1 antibody, Rabbit polyclonal	Sigma-Aldrich	Cat#07645; RRID:AB_310773

**Chemicals, peptides, and recombinant proteins**		

Tamoxifen	MP Biomedicals	Cat#215673883
TrypLE™ Express Enzyme	Fisher Scientific	Cat#12605010

**Critical commercial assays**		

RNeasy Mini Plus Kit	QIAGEN	Cat#74136
QIAshredder	QIAGEN	Cat#79656
RNA*later*® Solution	Fisher Scientific	Cat#AM7023
Lipofectamine™ LTX Reagent	Invitrogen	Cat#15338100
Lipofectamine™ 3000 Transfection Reagent	Invitrogen	Cat#L3000015
qScript™ XLT One-Step RT-qPCR ToughMix®, Low ROX™	QuantaBio	Cat#95134
PerfeCTa® qPCR FastMix® II, Low ROX™	QuantaBio	Cat#95120
Phusion Site-Directed Mutagenesis Kit	Thermo Scientific	Cat#F-541

**Deposited data**		

RNA-seq raw data files	NCBI Gene Expression Omnibus	GEO: GSE229698

**Experimental models: Cell lines**		

Human Umbilical Vein Endothelial Cells (HUVEC)	Fisher Scientific	Cat#C0035C; RRID:CVCL_2959
293 human embryonic kidney cell line	Sigma-Aldrich	Cat#85120602
COS-1 African green monkey kidney cell line	Sigma-Aldrich	Cat#88031701

**Experimental models: Organisms/strains**		

Mouse: *Traf7*^*+/fl*^ on C57BL/6 background	This paper	N/A
Mouse: B6.FVB-Tg(EIIa-cre)C5379Lmgd/J	The Jackson Laboratory	JAX: 003724; RRID:IMSR_JAX:003724
Mouse: B6.Cg-Tg(Tek-cre)12Flv/J	The Jackson Laboratory	JAX: 004128; RRID:IMSR_JAX:004128
Mouse: C57BL/6-Tg(Cdh5-cre/ERT2)1Rha	Taconic	Model: 13073

**Oligonucleotides**		

MISSION® esiRNA targeting human TRAF7	Sigma-Aldrich	EHU071571-20UG
MISSION® esiRNA targeting human SCRIB	Sigma-Aldrich	EHU141131-20UG
MISSION® esiRNA targeting human MAP3K3	Sigma-Aldrich	EHU101361-20UG
MISSION® esiRNA targeting human MAP2K3	Sigma-Aldrich	EHU088281-20UG
MISSION® esiRNA targeting RLUC	Sigma-Aldrich	EHURLUC-20UG
Primers for *Traf7* mice genotyping by conventional PCR (PCR product: WT allele 242 bp, floxed allele 375 bp): T7-Fwd: GCCATAGGGACATTTAACCTGCA T7-Rev: CTAATGCCATACTGCAGACCCA	This paper	N/A
TaqMan assay for genotyping of the WT *Traf7* mouse allele by real-time PCR: WT-3’-Fwd: CCTTGGGCTTAACTCTGCTTAT WT-3’-Rev: CACCCAGAAAGTCCAACAAGA WT-3’-Probe: CAGCCTTGGGAAGAGCCTACTGAC	This paper	N/A
TaqMan assay for genotyping of the floxed *Traf7* mouse allele by real-time PCR: Flox-3’-Fwd: GCTAGTACCTTGGGCTTAACTC Flox-3’-Rev: TGTTCGAACGAAGTTCCTATTCT Flox-3’-Probe: ACGGACACAATCCCACGAACGTAC	This paper	N/A
TaqMan assay for genotyping of the knockout *Traf7* mouse allele by real-time PCR: KO-Fwd: GAGCTAGTTGGCTTCCTCAAA KO-Rev : GGGAAGAGCCTACTGACAGATTA KO-Probe: TGGTACCGTACGTGTAGAAGCTACCA	This paper	N/A
TaqMan assay for mouse *GAPDH* RT-qPCR: GAPDH-Fwd: CCTGTTGCTGTAGCCGTATT GAPDH-Rev: AACAGCAACTCCCACTCTTC GAPDH-Probe: TTGTCATTGAGAGCAATGCCAGCC	This paper	N/A
TaqMan assay for mouse *KLF2* RT-qPCR: KLF2-Fwd: GGCAAGACCTACACCAAGAG KLF2-Rev: TCCTTCCCAGTTGCAATGAT KLF2-Probe: CGTACACACACAGGTGAGAAGCCT	This paper	N/A
TaqMan assay for mouse *KLF4* RT-qPCR: KLF4-Fwd: TTTCCTGCCAGACCAGATG KLF4-Rev: CTTTGGCTTGGGCTCCT KLF4-Probe: TTATCAAGAGCTCATGCCACCGGG	This paper	N/A
TaqMan assay for mouse *PECAM1* RT-qPCR: PECAM1-Fwd: GTGGTCATCGCCACCTTAATA PECAM1-Rev: TTCTCGCTGTTGGAGTTCAG PECAM1-Probe: AAAGCCAAGGCCAAACAGAAACCC	This paper	N/A

**Recombinant DNA**		

p3XFLAG-CMV™-7.1 Expression Vector	Sigma-Aldrich	Cat#E7533
p3XFLAG-CMV™-7-BAP Control Plasmid	Sigma-Aldrich	Cat#C7472
Human TRAF7 (NM_032271) ORF Clone in pcDNA3.1^+^/C-(K)DYK	GenScript	Cat#OHu30808D
SCRIB (H. sapiens) in pANT7_cGST	DNASU Plasmid Repository	CloneID: HsCD00731633
RFWD2 (H. sapiens) in pANT7_cGST	DNASU Plasmid Repository	CloneID: HsCD00639431
pCMV-HA	Clontech	Cat#631604
mCh-Climp63	AddGene	Cat#136293; RRID:Addgene_136293
pD2EGFP-N1	This paper	N/A
p3XFLAG-CMV™-7.1-COP1	This paper	N/A
p3XFLAG-CMV™-7.1-TRAF7	This paper	N/A
p3XFLAG-CMV™-7.1-TRAF7-N520S	This paper	N/A
p3XFLAG-CMV™-7.1-TRAF7 (1-670)	This paper	N/A
p3XFLAG-CMV™-7.1-TRAF7 (1-384)	This paper	N/A
p3XFLAG-CMV™-7.1-TRAF7 (1-292)	This paper	N/A
p3XFLAG-CMV™-7.1-TRAF7 (1-127)	This paper	N/A
p3XFLAG-CMV™-7.1-TRAF7 (121-292)	This paper	N/A
p3XFLAG-CMV™-7.1-TRAF7 (281-670)	This paper	N/A
p3XFLAG-CMV™-7.1-TRAF7 (375-670)	This paper	N/A

**Software and algorithms**		

GraphPad Prism 9	GraphPad Software	Prism - GraphPad
HISAT2 (v2.0.5)	SciCrunch Registry	RRID:SCR_015530
featureCounts (v1.5.0-p3)	SciCrunch Registry	RRID:SCR_012919
DESeq2 R package (v1.20.0)	SciCrunch Registry	RRID:SCR_012823
edgeR (v3.22.5)	SciCrunch Registry	RRID:SCR_012802
GATK (v4.1.1.0)	SciCrunch Registry	RRID:SCR_001876
rMATS (4.1.0)	SciCrunch Registry	RRID:SCR_023485
STAR-Fusion (v1.9.0)	Novogene	https://www.novogene.com/
clusterProfiler	SciCrunch Registry	RRID:SCR_016884
STAR	SciCrunch Registry	RRID:SCR_004463

### Resource availability

#### Lead contact

Further information and requests for reagents should be directed to and will be fulfilled by the lead contact, Ian F. Dunn (ian-dunn@ouhsc.edu).

#### Materials availability

Plasmids and genetically modified mice generated in this study are available from the lead contact upon request.

### Experimental model and study participant details

#### *Traf7-*deficient mice

All housing and experimental use of mice were carried out in AAALAC-accredited facility in accordance with United States federal, state, local, and institutional regulations and guidelines governing the use of animals and were approved by OUHSC Institutional Animal Care and Use Committee. *Traf7* targeting vector and *Traf7*^*+/fl*^ mice on C57BL/6 background were generated by the Ingenious Targeting Laboratory (Ronkonkoma, NY). To generate *Traf7*^*+/-*^ mice, *Traf7*^*+/fl*^ mice were crossed with *E2a-Cre*-trangenic animals purchased from the Jackson Laboratories (B6.FVB-Tg(EIIa-cre)C5379Lmgd/J; JAX stock #003724). To generate endothelium-specific knockout mice, *Traf7*^*+/fl*^ mice were crossed with *Tie2-Cre*-transgenic animals purchased from the Jackson Laboratories (B6.Cg-Tg(Tek-cre)12Flv/J; JAX stock #:004128). Subsequently, *Traf7*^*+/fl*^*·Tie2-Cre*-transgenic mice were crossed with *Traf7*^*fl/-*^ or *Traf7*^*fl/fl*^ animals. For conditional deletion of *Traf7* in postnatal endothelium, *Traf7*^*+/fl*^ mice were crossed with *Cdh5(PAC)-CreERT2*-trangenic animals purchased from Taconic (C57BL/6-Tg(Cdh5-cre/ERT2)1Rha; Model #:13073). Subsequently, *Traf7*^*+/fl*^*·Cdh5(PAC)-CreERT2*-transgenic mice were crossed with *Traf7*^*fl/-*^ or *Traf7*^*fl/fl*^ animals. Embryos and neonates were used strictly based on their genotype without any exclusion of males or females.

#### Cell lines

All cell lines were cultured in 37°C incubator with a humidified atmosphere of 5% CO_2_ in air. Human umbilical vein endothelial cells (HUVECs) were purchased from (ThermoFisher, C0035C) and cultured in Human Large Vessel Endothelial Cell Basal Medium (ThermoFisher, M-200-500) supplemented with Large Vessel Endothelial Supplement (LVES) (ThermoFisher, A1460801) in the absence of antibiotics and antimycotics. Human embryonic kidney cell line 293 (HEK293) (Sigma-Aldrich, 85120602) and COS1 (Sigma-Aldrich, 88031701) were cultured in Gibco™ EMEM (Fisher Scientific, 67-008-6) supplemented with10% FCS, 2 mM glutamine, and 1% non-essential amino acids.

### Method details

#### Mouse genotyping

Conventional or real-time PCR of genomic DNA from embryonic stem cells, tail DNA, or yolk sacs were performed with primer sets listed in the key resources table. The Cre gene genotyping was done as described on the web sites of Jackson Laboratories and Taconic.

#### Southern blot genotyping

Mouse tail DNA was analyzed by Southern blot. Genomic DNA was isolated, digested with *Sca I*, and resolved in 0.8% agarose gel. Membranes were hybridized with a 750-bp long 5′ probe generated from genomic DNA by PCR using primers (Forward: GATATCTGTCTCATCTCCCCTCCT and Reverse: GGTAGAGTAATAATGACCATGGGC) and cloned into pCR-TOPO vector. The bands of 26.3 Kb, 7.9 Kb, and 17.9 Kb correspond to WT, Floxed, and KO alleles, respectively (Figure S1C).

#### Embryological analysis

To harvest embryos at specified embryologic stages, timed pregnancies were set up. The embryos were considered 0.5 days post coitus (dpc) at noon of the day of detection of the vaginal plug. On E9.5 and E10.5, females were euthanized and embryos extracted. After removal from the uterus, yolk sacs and embryos were examined for gross abnormalities. Embryonic genotyping was done on genomic DNA purified from yolk sacs. Whole embryo images were obtained at total magnifications of 15x and 45x (combination of magnifications of 1.5x and 4.5x objective lens with 10x ocular lens) using an AmScope microscope with a MU1003 digital camera and AmScope software (AmScope). Embryos were fixed in 4% paraformaldehyde solution in PBS, embedded in paraffin and sagittal 5 μm sections were cut and stained with hematoxylin and eosin.

#### CD31 staining of embryos

CD31 staining of embryos was done as described in.^30^ Briefly, embryos were fixed in 4% paraformaldehyde in PBS overnight at 4°C, rinsed with PBS, dehydrated in methanol, bleached with 5% H_2_O_2_ in methanol for 5 hours, and then rinsed and stored in methanol. After rehydration, embryos were blocked and then incubated with 1:200 dilution of rat anti-mouse platelet endothelial cell adhesion molecule-1 (PECAM-1) (Fisher Scientific, BDB557355). After washing embryos were then incubated with secondary antibody of goat anti-rat immunoglobulin G (IgG) conjugated horseradish peroxidase (Fisher Scientific, OB3010-05). Horseradish peroxidase detection was done by using Metal Enhanced DAB Substrate Kit (Fisher Scientific, PI34065).

#### RNA-seq and differential expression (DE) analysis

E9.5 embryos were extracted from yolk sacs and immediately placed in RNA*later*® Solution (Fisher Scientific, #AM7023). They were kept at 4°C for 24 hours and transferred to -80°C for long-term storage before RNA extraction. Total RNA was extracted using the RNeasy Plus Micro Kit (QIAGEN, #74034) and QIAshredder (QIAGEN, #79656) according to the manufacturer’s instructions. Preparation of cDNA libraries, sequencing and analysis were conducted by Novogene Co., LTD (Beijing, China). Significant DEGs were defined as those that had both an absolute log2 fold change ≥ 1 as well as a false discovery rate adjusted p-value ≤ 0.05 for each comparison independently.

#### Quantitative PCR (RT-qPCR)

Total cell RNA was used to measure gene mRNA levels by real-time qPCR. Reverse transcription and cDNA amplification were performed in one tube using qScript™ XLT One-Step RT-qPCR ToughMix®, Low ROX™ (VWR Quanta Biosciences™, #95134) on an Applied Biosystems 7500 Fast Real-Time PCR System (Fisher Scientific). Sample reactions were run in 3-6 replicates. Each mRNA analysis was run in a DuPlex PCR reaction with *Gapdh* as an internal control. Standard curves for each gene were run to verify the linear range of amplification. Input RNA was kept under 200 ng per reaction to stay within the linear range for *Gapdh* levels. All data were analyzed in Microsoft Excel with the built-in analysis methods. TaqMan assays used for RT-qPCR are listed in the key resources table.

#### Brain hemorrhage analysis

Tamoxifen feeding of neonatal pups was done as described in.^58^ Tamoxifen was dissolved in sterile corn oil at a concentration of 10 mg/ml and administered to neonatal *Traf7*^*+/fl*^*·Cdh5(PAC)-CreERT2* and *Traf7*^*fl/-*^*·Cdh5(PAC)-CreERT2* pups by feeding to the mouth starting at P1. The tamoxifen feeding schedule was: P0–2, 5 mg per day; P3–5, 7.5 mg per day; after P6, 10 mg per day. Brains were dissected at P13, fixed overnight in 4% PFA, washed in PBS, and dehydrated in 100% EtOH. After that, they were embedded in paraffin and coronal 5 μm sections were cut and stained with hematoxylin and eosin.

#### siRNA constructs and transfections

All mission® esiRNA constructs (RLUC, EHURLUC-20.00UG; MAP3K3, EHU101361-20.00UG; MAP2K3, EHU088281-20.00UG; SCRIB, EHU141131-20.00UG; TRAF7, EHU071571-20.00UG) were purchased from Sigma-Aldrich. For shear stress test we used HUVEC cells between passages 3 and 5. Cells were seeded at 5x10^5^ of cells in 2 ml of medium into each well of two six-well cell culture dishes. Thirty-six hours later cells were transfected usingInvitrogen™ Lipofectamine™ RNAiMAX Transfection Reagent (Fisher Scientific, 13-778-075). Next morning, one plate was left under static conditions, while another was shaken for 6 hours at 150 rpm on a horizontal orbital shaker system Shaker, Extreme (Ohaus, SHEX1619DG).

#### Other cell cultures, plasmids, and transfections

HEK293 (Sigma-Aldrich, 85120602) and COS1 (Sigma-Aldrich, 88031701) cell lines were cultured in Gibco™ EMEM (Fisher Scientific, 67-008-6) supplemented with 10% FCS, 2 mM glutamine, and 1% non-essential amino acids. Human TRAF7 cDNA in expression vector pcDNA3.1+/C-(K)-DYK (NM_032271) was purchased from GenScript. Plasmid p3xFLAG-CMV7.1 (E4026) and control construct p3xFLAG-CMV7.1-BAP (C7472) were purchased from Sigma-Aldrich. Expression plasmids for FLAG-tagged human COP1, SCRIB and TRAF7 and its mutants were constructed by PCR amplification of the corresponding cDNA fragments and subsequent cloning into p3xFLAG-CMV7.1 vector containing an N-terminal 3xFLAG tag. Expression plasmids for mCherry-tagged TRAF7 and its mutants were also constructed by PCR amplification of mCherry open reading frame and subsequent cloning into p3xFLAG-CMV7.1-TRAF7 constructs. Expression plasmids for EGFP-tagged SCRIB were also constructed by PCR amplification of EGFP open reading frame and subsequent cloning into p3xFLAG-CMV7.1-TRAF7 constructs. A Phusion Site-Directed Mutagenesis Kit (Thermo Scientific, F-541) was used to introduce a point mutation for N520S amino acid change in p3xFLAG-CMV7.1-TRAF7 construct. Transient transfections were carried out using Invitrogen™ Lipofectamine™ LTX Reagent with PLUS™ Reagent (Fisher Scientific, 15-338-100) according to the manufacturer’s instructions. Not more than 2 μg of total plasmid DNA per 10^6^ cells were used.

#### Cell lysis and co-immunoprecipitations

Whole embryos and HUVECs were lysed in 1x Laemmli Sample Buffer (BIO-RAD, 1610747) with 100 mM NaCl, 50 mM DTT, and 1 μg/ml phosphatase inhibitor cocktail 3 (SigmaAlrich, P0044) mixed with glass beads, and shaken in an Eppendorf shaker at 2,000 RPM at 85°C for 10 min. For co-immunoprecipitations HEK293 cells were washed in 1xPBS and lysed in buffer containing 20 mM Tris-HCl pH 7.5, 20 mM NaCl, 1x Thermo Halt protease inhibitors, 0.5% IGEPAL CA-630, 1 mM DTT, 1 mM EDTA, and 0.1 mM MG-132. Lysates were sonicated on ice using Fisher Scientific FB120 at 5 min/10 sec/10 sec, rotated with M2-Magnetic beads (Sigma-Alrich, M8823) for 3 hours and washed 3 times with 20 mM Tris-HCl pH 7.5, 20 mM NaCl, 0.1% IGEPAL CA-630, and 1 mM EDTA.

#### Western blot analysis

Samples were run on an 4-15% Mini-PROTEAN® TGX™ Protein Gel (BIO-RAD, 4561083); transferred to nitrocellulose membranes (BIO-RAD, 1620145), which were blocked with Blotting-Grade Blocker (BIO-RAD, 1706404); and probed with antibodies from CellSignaling: MEKK3 (5727), MEKK2 (5727), MARK2 (9118), ERK5 (3552), MEK5 (40737), and SCRIB (4475). Sp1 antibody (Sigma Aldrich, 07-645), and α-tubulin (3873) were used as loading controls. Secondary antibodies included goat anti-rabbit IgG, HPR-linked (CellSignaling, 7074) and goat anti-mouse-HRP Conjugate (BIO-RAD, 1705047). Blots were developed with chemiluminescent Western blotting substrates: SuperSignal™ West Femto (ThermoFisher, PI34094) or Pierce™ ECL Western blotting substrate (ThermoFisher, 32209).

#### Mass spectrometry analysis

Mass spectrometry analysis was done as previously described.^90^ Briefly, after samples were run on a gel, the lanes were subdivided into narrow segments, which were cut from the gel. Each gel segment was washed, destained, lyophilized, rehydrated and digested with trypsin overnight at 37°C. The peptides were extracted and run on Finnigan LCQ DUO Thermo Quest Ion Trap Mass Spectrometer with Protana nanospray ion source interfaced to Phenomenex Jupiter 10 μm C18 reverse-phase capillary column. The resulting MS/MS spectra were analyzed using Sequest software.

### Quantification and statistical analysis

Statistical analysis of progeny Mendelian distribution and brain hemorrhage was analyzed using the Chi-squared test (Graphpad Prism 9.5.1). Quantification of the embryonic vascular network was performed using Angiogenesis Analyzer in ImageJ software. Number of branching points (junctions) and segments were analyzed within a fixed-size window in at least three different embryos of each genotype. In RNA-seq DE analysis, differential expression was calculated using the Wald test implemented in the R package DESeq2. Significantly differentially expressed genes were defined as those that had both an absolute log2 fold change ≥ 1 as well as a false discovery rate (FDR) adjusted p-value ≤ 0.05 for each comparison independently. Gene expression levels by RT-qPCR for all genes of interest were calculated by comparative ΔC_T_ experiment runs on AB7500 Fast machine and analyzed using the 7500 Software v2.3. The ΔC_T_ data for each gene of interest were normalized on *Gapdh* ΔC_T_ data. Statistical analyses of RT-qPCR data were performed and plotted using Excel with the built-in analysis methods.

## Data Availability

• RNA-seq raw data files have been deposited on NCBI Gene Expression Omnibus and are publicly accessible (GEO Accession viewer (nih.gov)). The GEO accession number is listed in the key resources table. • This paper does not report original code. • Any additional information required to reanalyze the data reported in this paper is available from the lead contact upon request.

## Supplemental information

Document S1. Figures S1–S6

Table S1. Transcriptome comparison of E9.5 *Traf7*^*GLOko*^ vs. WT embryos by RNA-seq. List of DEGs, related to Figure 3

Table S2. Transcriptome comparison of E9.5 *Traf7*^*ECko*^ vs. *Traf7*^*EChet*^ embryos by RNA-seq. List of DEGs, related to Figure 4
