# Supplementary material for: TRAF7 is an essential regulator of blood vessel integrity during mouse embryonic and neonatal development
Source: iScience. 2023 Jul 25;26(8):107474. doi: 10.1016/j.isci.2023.107474 (PMC10424150; doi:10.1016/j.isci.2023.107474)
Supplement: Document S1. Figures S1–S6 [file mmc1.pdf]

iScience, Volume *26*

Supplemental information

TRAF7 is an essential regulator of blood vessel integrity during mouse embryonic and neonatal development

Erdyni N. Tsitsikov, Khanh P. Phan, Yufeng Liu, Alla V. Tsytsykova, Mike Kinter, Lauren Selland, Lori Garman, Courtney Griffin, and Ian F. Dunn

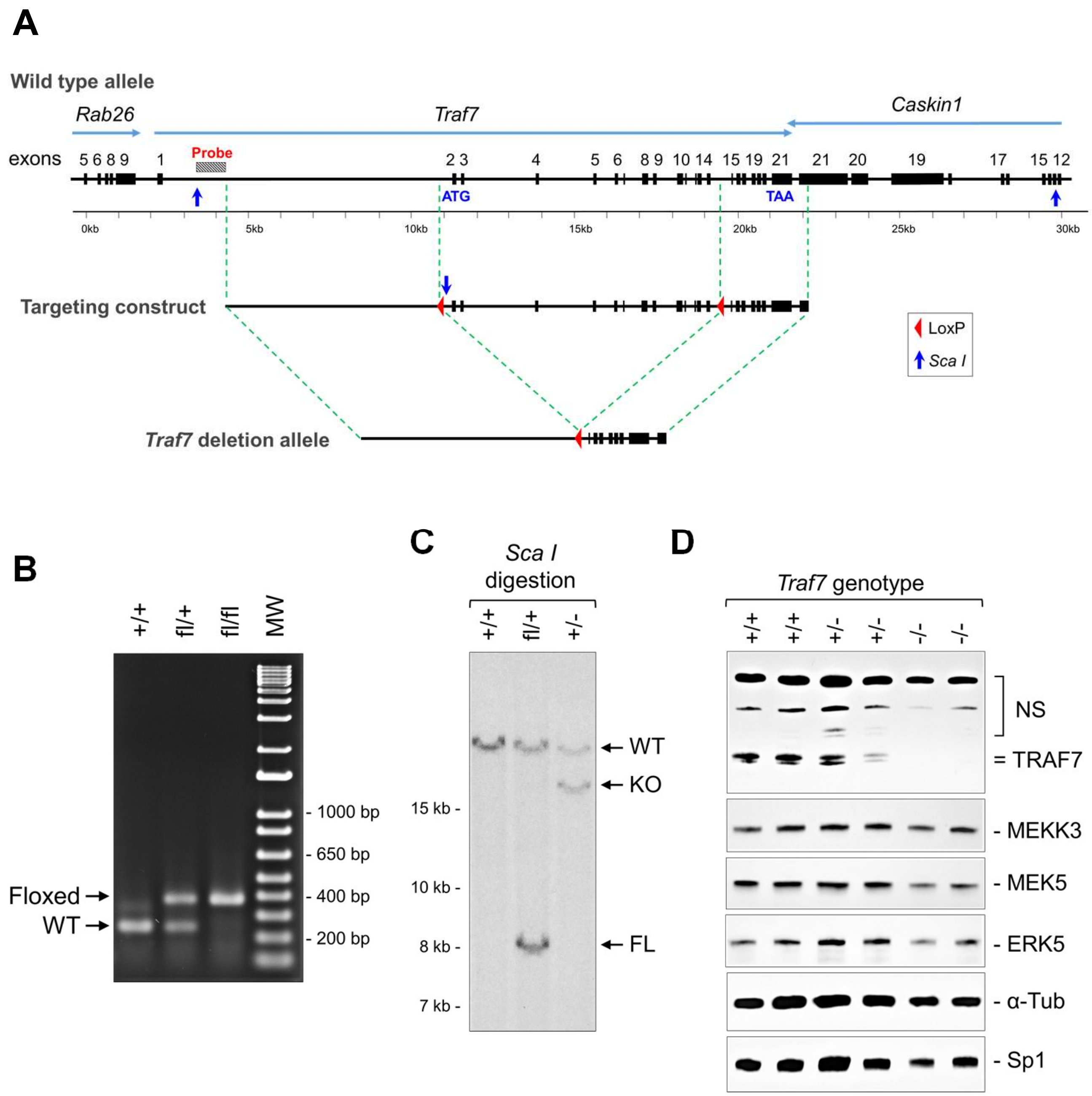

Figure S1. Targeted deletion of Traf7 gene.
(**A**) Schematic drawing (to-scale) of the *Traf7* gene and part of the surrounding locus on mouse chromosome 17. DNA is shown as a black line. Exons are represented as black boxes and numbered. Directions of gene transcription are indicated by blue arrows above. LoxP sites are shown as red triangles. *Sca I* restriction sites used in Southern blot analysis are marked by blue arrows. Maps of "Targeting construct" and the resulting "*Traf7* deletion allele" are positioned below the "Wild type allele" with corresponding dotted green lines illustrating the Cre-mediated *Traf7* gene excision process. (**B**) Detection of WT and *Traf7*-floxed alleles by conventional PCR and agarose gel electrophoresis. (MV): Molecular Weight markers (1 Kb Plus Ladder). (**C**) Southern blot analysis of genomic DNA purified from WT (+/+), *Traf7*$^{fl/+}$ (fl/+), and *Traf7*^{GLOhet} (+/-) mouse tails, digested with *Sca I*, and probed with region spanning the genomic region from intron 1 of the *Traf7* gene immediately outside of the targeting construct as shown in red font in (A). (**D**) Western blot analysis of TRAF7, MEKK3, MEK5, and ERK5 protein expression in WT (+/+), *Traf7*^{GLOhet} (+/-), and *Traf7*^{GLOko} (-/-) E9.5 whole embryo lysates. Two different embryos of each genotype were processed independently. Anti-α-Tubulin and anti-Sp1 antibodies were used as loading controls. (NS): non-specific bands. Related to Figure 1.

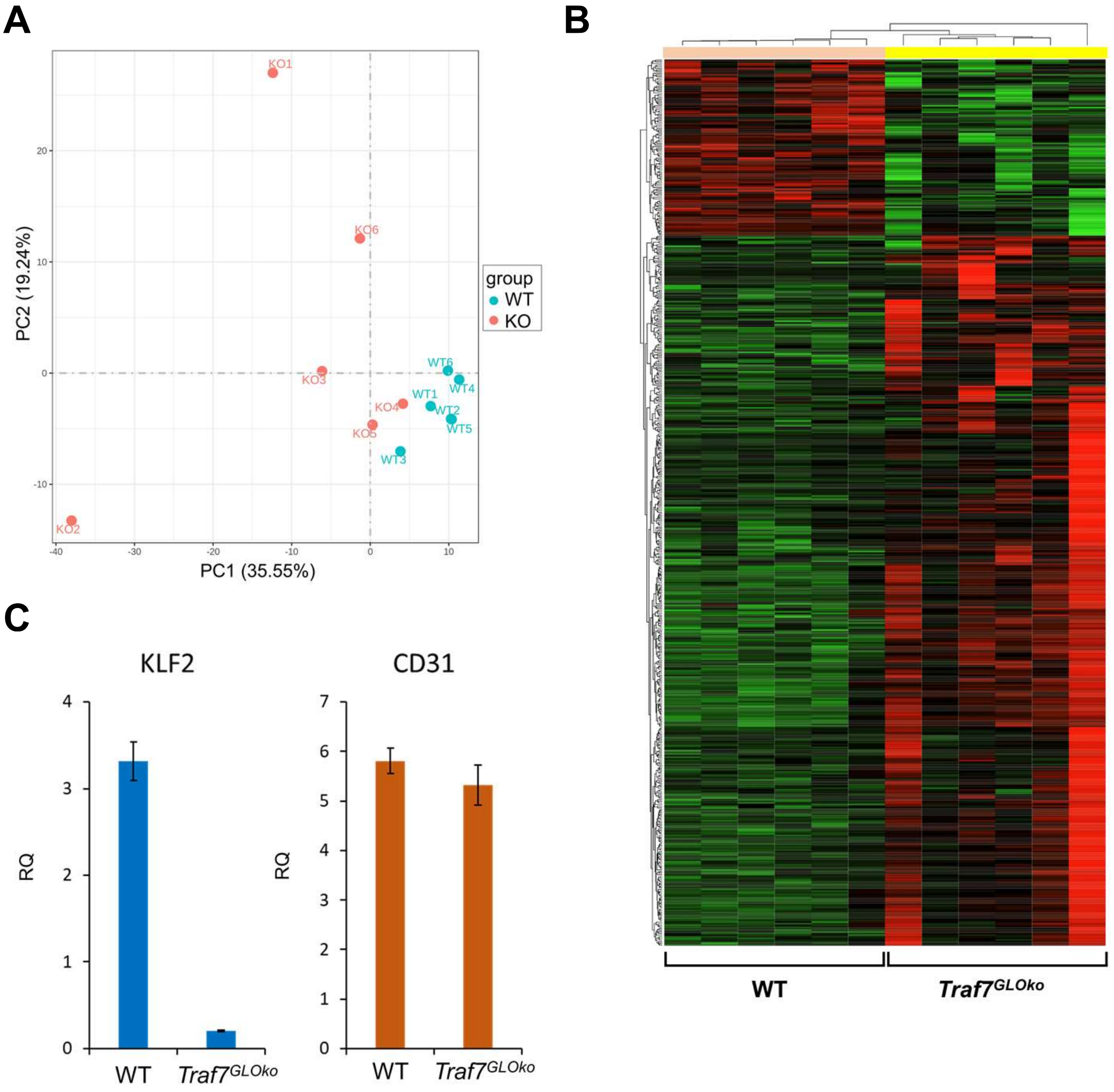

Figure S2. Differential gene expression in WT and *Traf7*-deficient mouse embryos.
(**A**) PCA Plot of RNA-seq analysis in WT and *Traf7*^{GLOko} (KO) embryos at E9.5. Each point corresponds to an individual embryo. (**B**) Heatmap of mRNA expression levels for all significant DEGs in WT versus *Traf7*^{GLOko} embryos by RNA-seq. (**C**) mRNA expression analysis of *Klf2* and *Cd31* in WT (n=6) versus *Traf7*^{GLOko} (n=8) embryos at E9.5. Total RNA was purified from whole mouse embryos and mRNA was amplified by RT-qPCR using *Gapdh* as internal control. Data are presented as relative quantity (RQ) Mean ± SEM. Related to Figure 3.

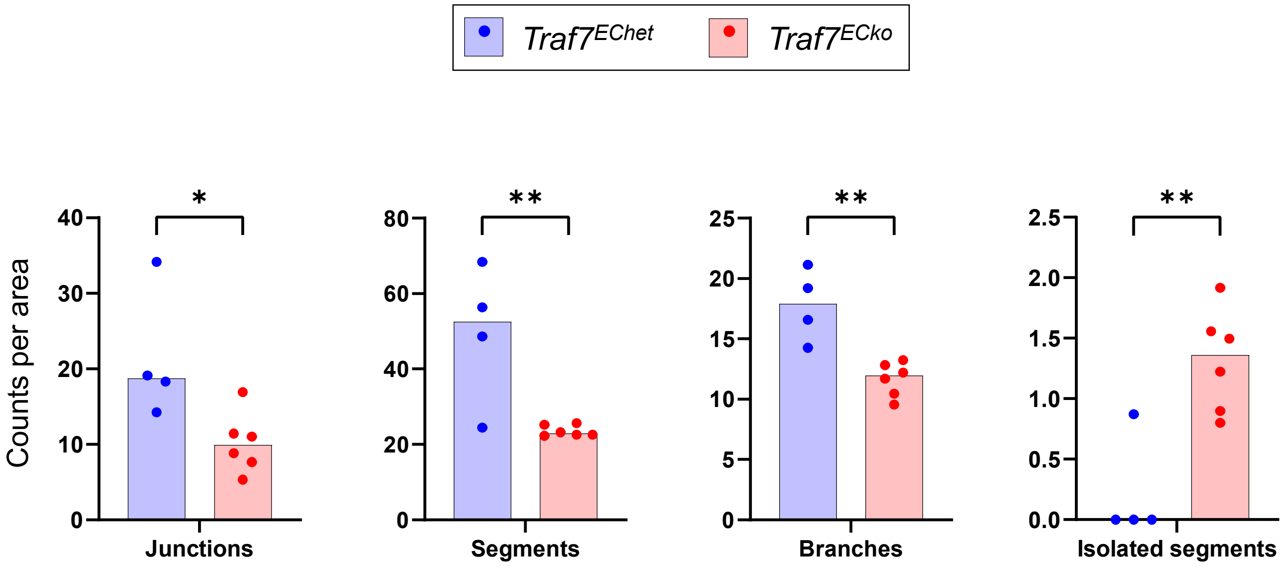

Figure S3. Quantification of the embryonic vascular defect in *Traf7*-deficient mouse embryos. Vascular network analysis was performed using Angiogenesis Analyzer in ImageJ software. Number of branching points (junctions), segments, and branches were analyzed within a fixed-size window in at least four different embryos of each genotype and plotted as the Median. (*) $p < 0.05$; (**) $p < 0.005$. Related to Figure 4C.

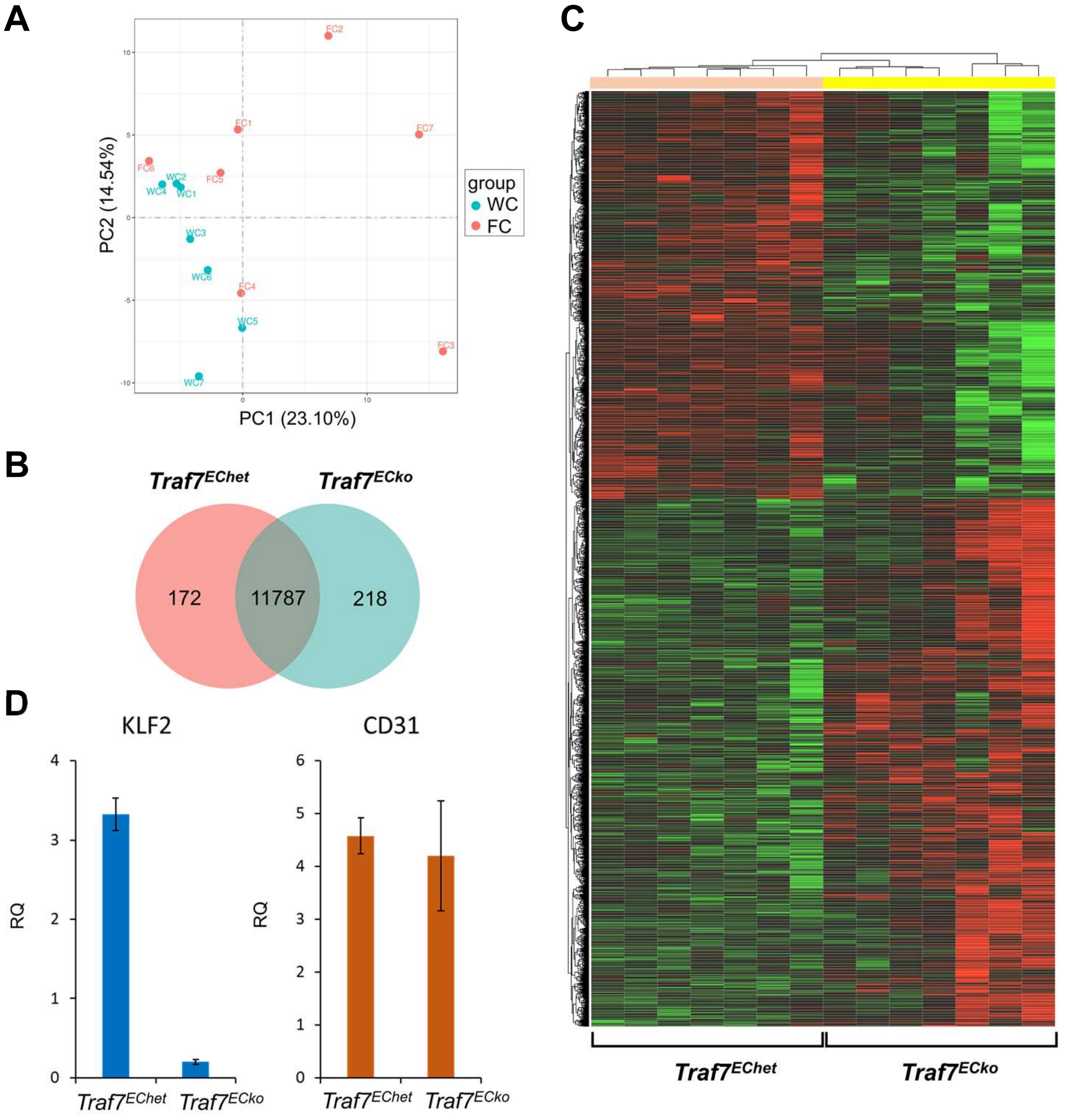

Figure S4. Altered gene expression in E9.5 embryos with endothelium-specific deletion of *Traf7*. (**A-C**) RNA-seq analysis of *Traf7*^{EChet} (WC) and *Traf7*^{ECko} (FC) embryos. (**A**) PCA Plot. Each point corresponds to an individual embryo. (**B**) Venn diagram showing DEGs overlap. (**C**) Heatmap of mRNA expression levels for all significant DEGs. (**D**) RT-qPCR mRNA expression analysis of *Klf2* and *Cd31* in WC (n=8) versus FC (n=7) embryos. *Gapdh* was used as an internal control. Data are presented as relative quantity (RQ) Mean ± SEM. Related to Figure 4D and E.

A

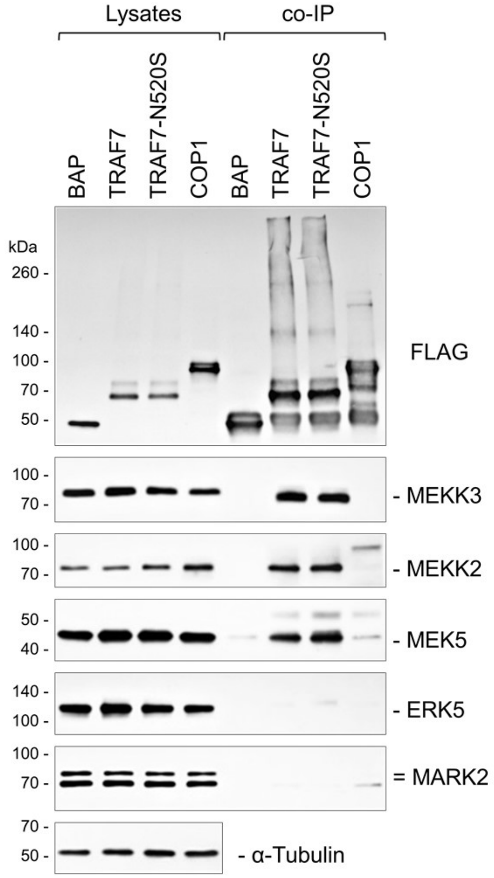

B

gene symbol	name	mascot score	protein mw	peptide match	sequence match
**SCRIB**	protein scribble homolog isoform a [Homo sapiens]	**1759**	178585	40	32
VBP1	prefoldin subunit 3 [Homo sapiens]	408	22783	11	8
NUDC	nuclear migration protein nudC [Homo sapiens]	274	38276	7	6
STUB1	E3 ubiquitin-protein ligase CHIP [Homo sapiens]	267	35290	7	6
PABPC1	polyadenylate-binding protein 1 [Homo sapiens]	241	70854	7	6
HNRNPA3	heterogeneous nuclear ribonucleoprotein A3 [Homo sapiens]	232	39799	11	6
HNRNPA0	heterogeneous nuclear ribonucleoprotein A0 [Homo sapiens]	220	30993	11	5
SNRNP200	U5 small nuclear ribonucleoprotein 200 kDa helicase [Homo sapiens]	216	246006	5	5

Figure S5. Association of TRAF7 with SCRIB.
(**A**) Western blot analysis of expression of several proteins and their co-immunoprecipitation (co-IP) with overexpressed FLAG-TRAF7 proteins using HEK293 whole cell lysates and indicated antibodies. Co-IP was done with anti-FLAG antibody. Anti-α-Tubulin antibody was used as the loading control. Blots shown are representative of at least four independent experiments. (**B**) Table showing top eight molecules associated with overexpressed TRAF7 in HEK293 by mass spectrometry analysis. Related to Figure 6.

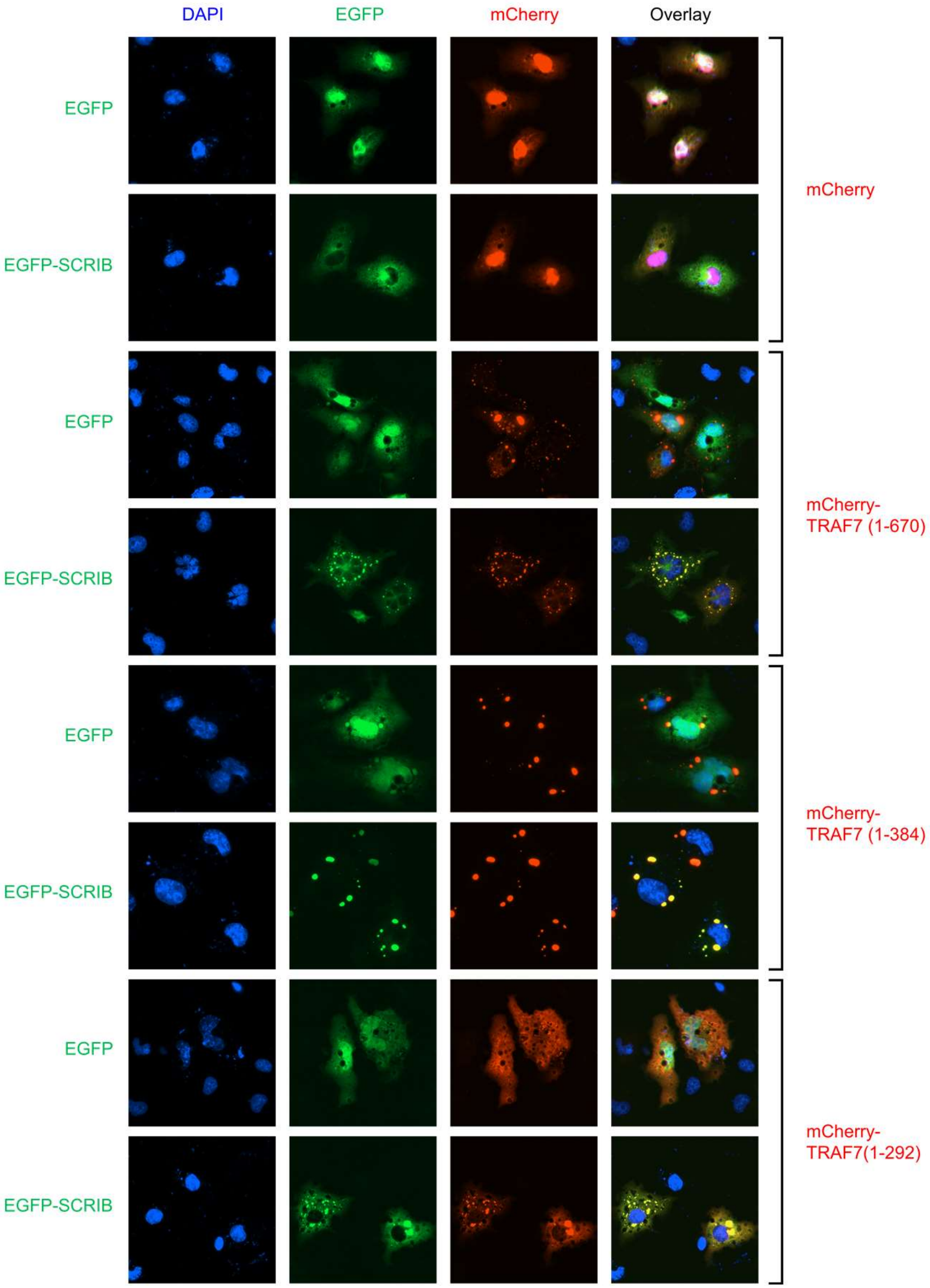

Figure S6. Association of TRAF7 with SCRIB.
Transient co-transfections of plasmids encoding SCRIB and full or truncated forms of TRAF7 as fluorescent fusion proteins in COS-1 cells line as indicated. Related to Figure 6.
